# Multi-horizon machine learning for population-level hypertension risk stratification in the UK Biobank

**DOI:** 10.1093/ehjdh/ztag128

**Published:** 2026-08-10

**Authors:** Akhil Naik, Ivan Olier, Ellen A Dawson, Garry McDowell, Deirdre A Lane, Gregory Y H Lip, Sandra Ortega-Martorell

**Affiliations:** Artificial Intelligence and Digital Technologies Research Institute, Liverpool John Moores University, Bryrom Street, Liverpool L3 3AF, UK; Liverpool Centre for Cardiovascular Science, University of Liverpool, Liverpool John Moores University and Liverpool Heart & Chest Hospital, Derby Street, Liverpool L7 8TX, UK; Artificial Intelligence and Digital Technologies Research Institute, Liverpool John Moores University, Bryrom Street, Liverpool L3 3AF, UK; Liverpool Centre for Cardiovascular Science, University of Liverpool, Liverpool John Moores University and Liverpool Heart & Chest Hospital, Derby Street, Liverpool L7 8TX, UK; Liverpool Centre for Cardiovascular Science, University of Liverpool, Liverpool John Moores University and Liverpool Heart & Chest Hospital, Derby Street, Liverpool L7 8TX, UK; Research Institute for Sport and Exercise Sciences, Liverpool John Moores University, Byrom Street, Liverpool L3 3AF, UK; Liverpool Centre for Cardiovascular Science, University of Liverpool, Liverpool John Moores University and Liverpool Heart & Chest Hospital, Derby Street, Liverpool L7 8TX, UK; School of Pharmacy and Biomolecular Sciences, Liverpool John Moores University, Byrom Street, Liverpool L3 3AF, UK; Liverpool Centre for Cardiovascular Science, University of Liverpool, Liverpool John Moores University and Liverpool Heart & Chest Hospital, Derby Street, Liverpool L7 8TX, UK; Department of Cardiovascular and Metabolic Medicine, Institute of Life Course and Medical Sciences, University of Liverpool, Derby Street, Liverpool L7 8TX, UK; Liverpool Centre for Cardiovascular Science, University of Liverpool, Liverpool John Moores University and Liverpool Heart & Chest Hospital, Derby Street, Liverpool L7 8TX, UK; Artificial Intelligence and Digital Technologies Research Institute, Liverpool John Moores University, Bryrom Street, Liverpool L3 3AF, UK; Liverpool Centre for Cardiovascular Science, University of Liverpool, Liverpool John Moores University and Liverpool Heart & Chest Hospital, Derby Street, Liverpool L7 8TX, UK

**Keywords:** Artificial intelligence, Multi-horizon prediction, Hypertension, Risk prediction, Explainability, Generative topographic mapping, UK Biobank

## Abstract

**Aims:**

Hypertension is a major contributor to cardiovascular morbidity and mortality, yet identifying individuals at risk before clinical diagnosis remains challenging. Here, we present a multi-horizon machine learning framework designed to model incident hypertension risk across multiple clinically meaningful time windows using data from 246 286 participants in the UK Biobank. The framework systematically compares predictive performance across five horizons under severe class imbalance, enabling analysis of how discrimination, precision, and risk drivers evolve as outcome prevalence changes over time.

**Methods and results:**

Seven classification algorithms were evaluated, including logistic regression, random forest, naïve Bayes, and four boosting-based ensemble methods. To enhance interpretability, we integrate SHapley Additive exPlanations (SHAP) with generative topographic mapping (GTM), combining feature-level attribution with population-level visualization of model predictions. Ensemble boosting models consistently achieved the strongest performance, with average precision increasing from 0.04 for the ≤2-year horizon to 0.22 for the ≤10-year horizon, while ROC-AUC remained relatively stable (∼0.75–0.79). Together, this framework reveals consistent predictors of hypertension risk (including baseline blood pressure, age, body mass index, medication burden, and cardiometabolic multimorbidity) and illustrates how combinations of risk factors organize hypertension risk across time horizons.

**Conclusions:**

Our results demonstrate how multi-horizon modelling and complementary explainability approaches can provide deeper insight into evolving disease risk patterns in large biomedical cohorts, supporting more interpretable and scalable strategies for population-level cardiovascular prevention. Such approaches may enable earlier identification of high-risk individuals and inform targeted screening and preventive interventions in routine care settings.

## Graphical Abstract

**Graphical Abstract:**
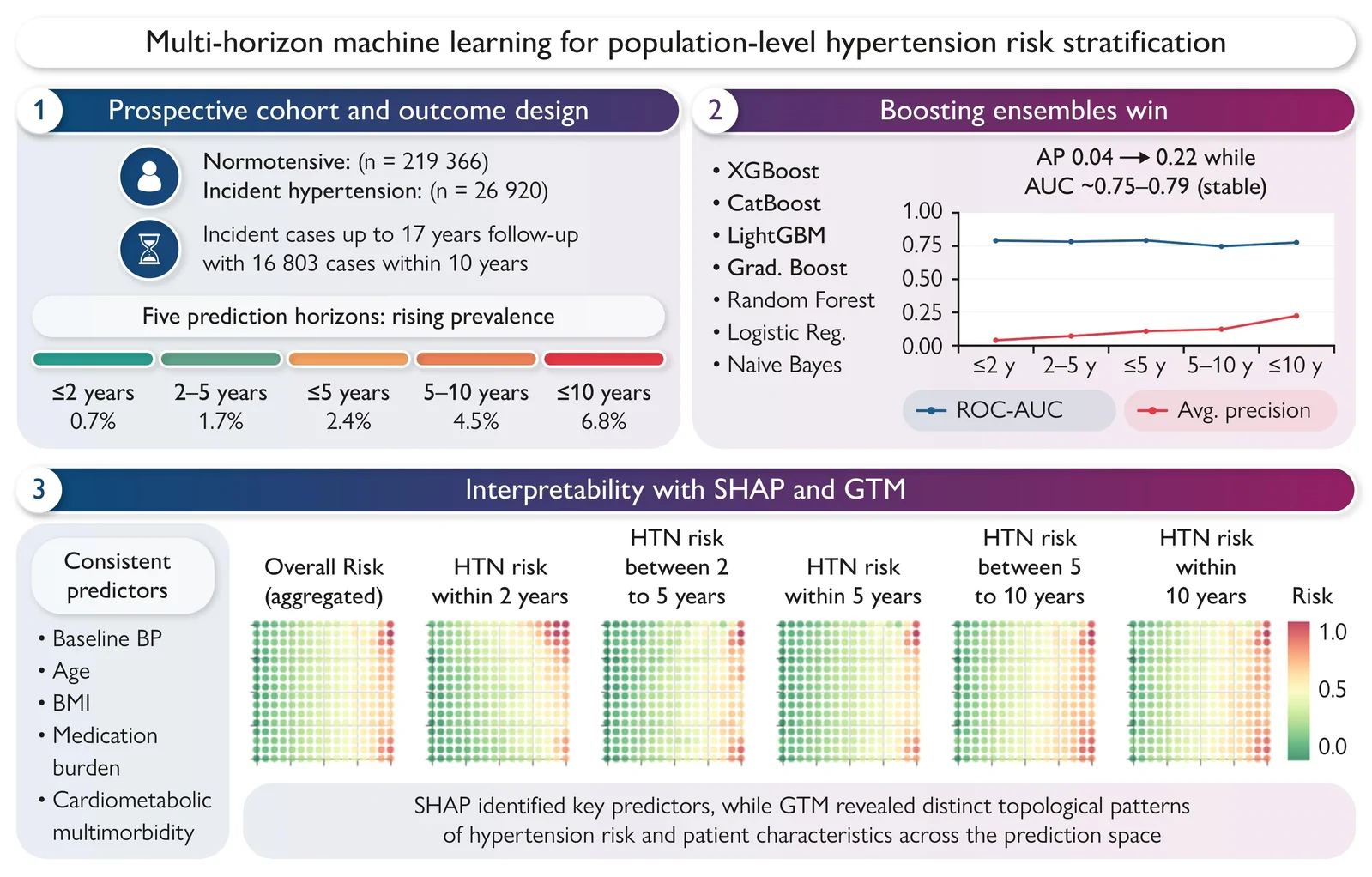

Highlights

- Multi-horizon machine learning framework predicts hypertension across five-time windows
- Boosting ensembles outperform baseline models in 246k UK Biobank participants
- Precision improves with longer horizons despite stable ROC-AUC (∼0.75–0.79)
- SHAP and GTM reveal population-level structures of hypertension risk

## Introduction

Hypertension (HTN) is a prevalent chronic cardiovascular condition characterized by sustained elevation of arterial blood pressure. Current European Society of Cardiology guidelines define hypertension as an office blood pressure >140/90 mmHg,^1^ whereas American Heart Association guidelines recommend lower thresholds (≥130/80 mmHg).^2^ Persistent elevation of blood pressure promotes endothelial dysfunction and vascular remodelling, increasing the risk of major cardiovascular complications, including myocardial infarction, stroke, kidney failure, and vascular dementia.^3,4^

Globally, hypertension remains a leading cause of premature mortality, accounting for an estimated 10.8 million deaths annually.^3^ Approximately 1.28 billion adults aged 30–79 years are affected worldwide, with nearly 46% of them unaware of their condition.^4^ In the UK, around 30% of adults have hypertension,^5^ and prevalence approaches 48% in the USA.^6^ These figures underscore the importance of effective prevention and earlier identification of individuals at risk. Advances in artificial intelligence (AI) and machine learning (ML) have expanded opportunities for large-scale, data-driven risk prediction in this context.^7^

Numerous models incorporating clinical, behavioural, socioeconomic, and genetic factors have been developed to predict incident hypertension.^8–11^ Historically, most studies have relied on conventional statistical approaches, including regression and survival analysis^12–14^; widely adopted tools such as the Framingham hypertension risk score exemplify this approach.^15–17^ Although interpretable and computationally efficient, these methods may zbe limited in capturing complex, non-linear interactions. Consequently, ML-based approaches have gained attention for their flexibility and scalability.^18^ However, many ML studies rely on cross-sectional designs,^11,19^ with fewer evaluating prospective cohorts,^11,20–25^ and very limited work systematically modelling longitudinal hypertension risk across multiple prediction horizons within large population cohorts.

The UK Biobank, a large prospective cohort with extensive phenotyping,^26^ offers an opportunity to address these gaps. Prior work by Zhang *et al*.^27^ applied a multitask Cox model to predict hypertension alongside other chronic diseases across multiple time horizons. However, the proportional hazards framework relies on linear covariate effects and proportional risk assumptions, which may constrain the modelling of complex, non-linear interactions among risk factors,^28^ motivating the development of more flexible ML frameworks capable of modelling non-linear interactions and heterogeneous risk trajectories in large longitudinal datasets.

Despite increasing use of explainable AI in healthcare, few hypertension studies integrate feature-level attribution with population-level visualization of prediction landscapes, limiting understanding of how combinations of risk factors structure disease risk across populations. Recent reviews highlight both the promise of AI-driven personalized risk prediction and persistent challenges related to class imbalance, interpretability, and clinical translation.^29^ In clinical practice, risk prediction is inherently time-dependent, with different decision thresholds and interventions relevant at short-, intermediate-, and long-term horizons.

Understanding how disease risk evolves across time horizons is essential for translating predictive models into preventive strategies. In this study, we propose and evaluate a multi-horizon machine learning framework for modelling incident hypertension risk using UK Biobank data. We model fixed-horizon risk across short-, intermediate-, and long-term windows and systematically evaluate predictive performance across horizons under severe class imbalance, using metrics appropriate for rare-event prediction. To enhance interpretability, we combine SHAP-based feature attribution with generative topographic mapping (GTM), enabling both feature-level explanation and population-level visualization of hypertension risk landscapes across time horizons.

## Results

### Data preparation

Among the total 502 128 UK Biobank participants retrieved, 204 816 had a valid recorded hypertension (HTN) diagnosis date in the UK Biobank records, irrespective of whether this occurred before, at, or after the initial assessment visit. Incident HTN events occurring after baseline were observed over a maximum follow-up period of 17 years (see Supplementary material  *S1*—Figure F2). Analyses were restricted to data collected at this initial/baseline assessment visit due to substantial attrition over time (see Supplementary material  *S1*—Figure F3), with participant attendance declining to 4.1% at the second, 18.3% at the third, and 2.8% at the fourth assessment visit. While facilitating multi-horizon risk prediction along with minimizing potential data bias, the five outcome variables created using the appropriate temporal groupings outlined earlier, showcased very low prevalences. The observed incident rates were 0.67% within 2 years, 1.69% between 2–5 years, 2.36% within 5 years, 4.46% between 5–10 years, and 6.82% within 10 years.

**Figure 1:**
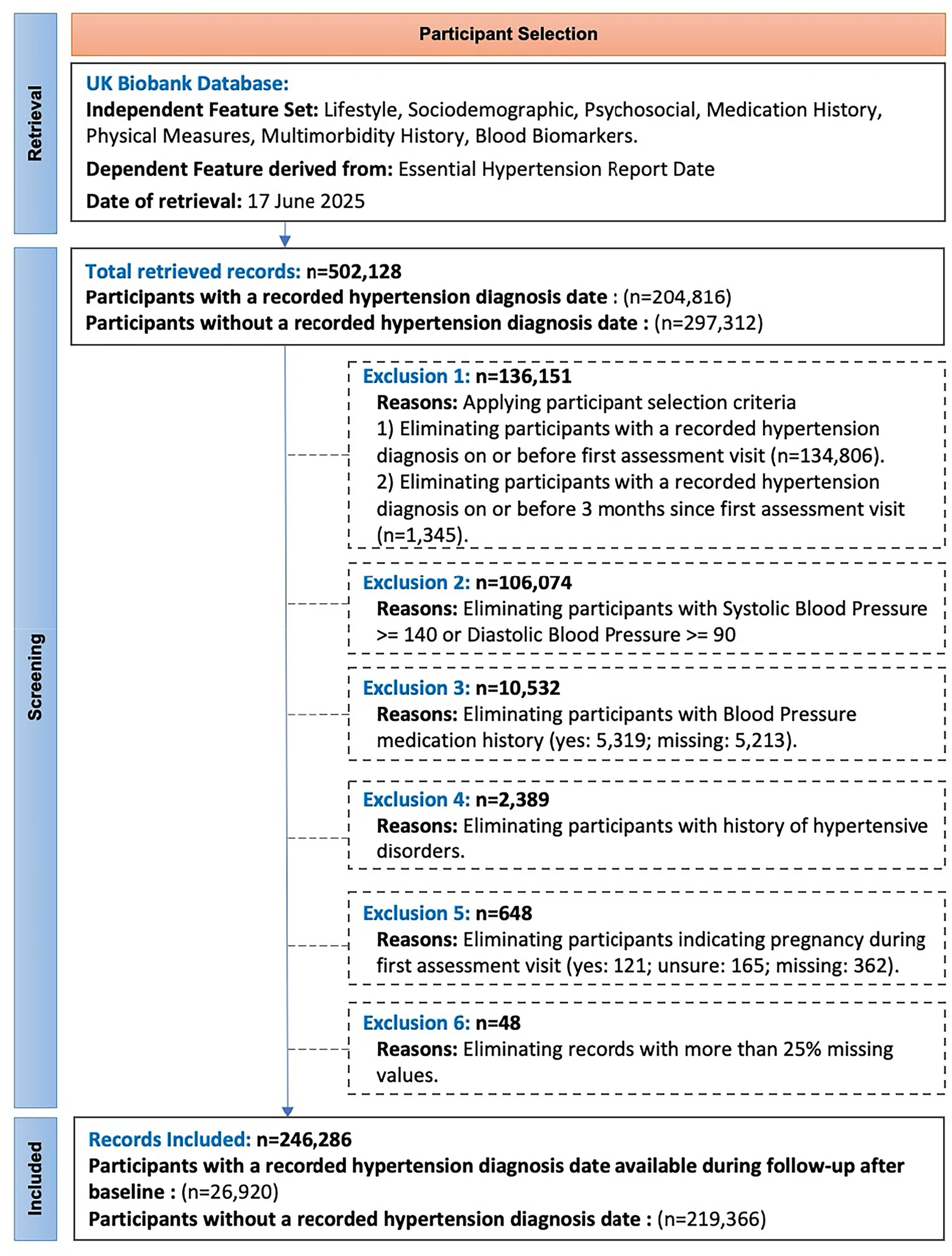
Screening and selection of participants for model development. Flow diagram illustrating participant selection from the UK Biobank, including sequential exclusion criteria applied at baseline and during preprocessing, with the number of records removed at each step and the composition of the final study cohort. Participants with incident hypertension correspond to those with a valid post-baseline hypertension diagnosis date recorded during follow-up.

A total of 246 286 participant records were included in the resulting analytical cohort, of which 219 366 participants had no recorded post-baseline hypertension diagnosis date in the available UK Biobank follow-up records. Records excluded, and the reasons for exclusion are detailed in *Figure 1*. Within this final cohort, 26 920 participants developed incident HTN during the full available follow-up period after baseline assessment (maximum follow-up of 17 years), corresponding to participants with a valid post-baseline HTN diagnosis date. Among these, 16 803 incident HTN cases occurred within 10 years.

A total of 131 independent features were retained for modelling, with eight features excluded due to high correlation (see Supplementary material—*S2*). Comprehensive descriptive statistics for all included variables, including demographic and clinical baseline characteristics of the analytical cohort, are provided in Supplementary material—*S3*.

**Figure 2:**
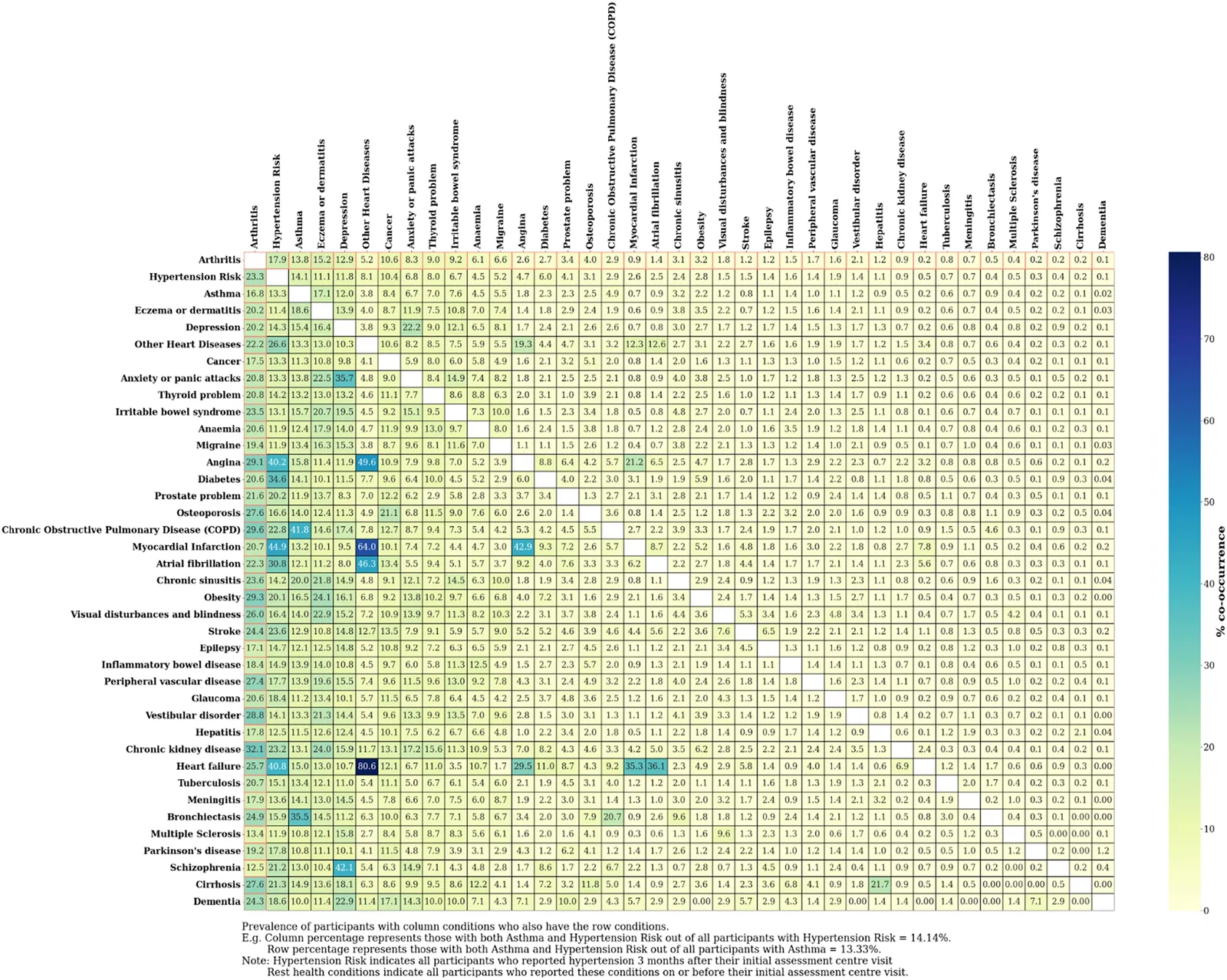
Multimorbidity co-occurrence heatmap for incident hypertension risk. Heatmap illustrating pairwise co-occurrence of chronic conditions included in the baseline multimorbidity profile used as candidate predictor variables. Cell values represent the percentage of participants with the row condition who also had the column condition. Colour intensity indicates the degree of co-occurrence. Hypertension risk refers to participants with a first recorded hypertension diagnosis after the initial assessment visit (baseline), excluding those with hypertension recorded on or before baseline or within three months of the initial assessment.

Multimorbidity history features were included among the baseline predictors used for model development because of their clinical relevance to hypertension risk prediction. Cardiovascular conditions were the most prevalent comorbidities associated with hypertension. *Figure 2* presents a descriptive heatmap of multimorbidity history, illustrating patterns of co-occurrence among conditions in the analytical cohort and contextualizing the multimorbidity information available to the models.

Data preprocessing identified the presence of outliers across multiple features; however, these observations were retained to preserve the representation of rare but clinically relevant profiles, given the substantial class imbalance in the dataset. Following preprocessing and data partitioning, the final analytical sample comprised 197 028 participant records allocated to model training and 49 258 participant records reserved for model testing.

### Model performance

We evaluated the performance of seven ML models across five binary classification tasks, each corresponding to a predefined temporal window for incident hypertension risk prediction (within 2 years, 2–5 years, within 5 years, 5–10 years, and within 10 years), using the performance metrics described in Section 4.5.

Across all prediction horizons, tree-based ensemble methods (XGB, LGBM, CATB, and GB) achieved higher performance than baseline classifiers (LR, NB, and RF). Among these models, CATB achieved the highest performance across multiple prediction tasks, while NB consistently produced the lowest performance. For all models, AUC values were generally higher for tree-based approaches compared with non-tree-based classifiers. Boosting-based models achieved higher AP scores than the bagging-based RF model. Differences in AP among the boosting models were small, with only marginal separation between the highest and second-highest performing algorithms. AP and AUC values for each model and prediction horizon are reported in *Tables 1* and *2*, respectively.

**Table 1:** Average precision score of respective models for each classification problem

Models	HTN risk within 2 years	HTN risk between 2–5 years	HTN risk within 5 years	HTN risk between 5–10 years	HTN risk within 10 years
**LGBM**	**0**.**0380**	0.0704	0.1064	0.1184	0.2172
**CATB**	0.0373	0.0694	**0**.**1076**	**0**.**1212**	**0**.**2233**
**XGB**	0.0371	**0**.**0708**	0.1066	0.1199	0.2203
**GB**	0.0340	0.0705	0.1064	0.1204	0.2225
**RF**	0.0333	0.0654	0.1009	0.1158	0.2113
**LR**	0.0332	0.0595	0.0905	0.1100	0.1959
**NB**	0.0300	0.0625	0.0913	0.0967	0.1800

**Table 2:** ROC-AUC score of respective models for each classification problem

Models	HTN risk within 2 years	HTN risk between 2–5 years	HTN risk within 5 years	HTN risk between 5–10 years	HTN risk within 10 years
**LGBM**	0.7889	0.7833	0.7949	0.7465	0.7717
**CATB**	0.7718	0.7846	0.7897	0.7458	0.7739
**XGB**	0.7954	0.7812	0.7940	0.7495	0.7734
**GB**	0.7793	0.7856	0.7914	0.7478	0.7746
**RF**	0.7867	0.7740	0.7824	0.7442	0.7660
**LR**	0.7671	0.7585	0.7647	0.7340	0.7556
**NB**	0.7088	0.7214	0.7200	0.6803	0.7009

The optimal hyperparameter configurations for each model and prediction horizon are provided in Supplementary Material  *S4*. Performance of the best-performing model for each prediction task is summarized in *Table 3*. AP values increased with longer prediction horizons, from 0.038 [95% confidence interval (CI): 0.030–0.053] for the 2-year prediction window to 0.223 (95% CI: 0.212–0.237) for the 10-year window. The optimal model varied across horizons: LGBM achieved the highest AP for prediction within 2 years, XGB performed best for the 2–5-year window, and CATB achieved the highest AP for predictions within 5 years and beyond.

**Table 3:** Optimal model scores for each classification problem

Hypertension Risk	Best Model	AP (95% C.I.)	AUC (95% C.I.)	Brier Score	Log Loss
Within 2 years	LGBM	0.0380	0.7889	0.0076	0.0479
		(0.0297, 0.0527)	(0.7652, 0.8127)		
Between 2–5 years	XGB	0.0708	0.7812	0.0210	0.1155
		(0.0615, 0.0841)	(0.7662, 0.7962)		
Within 5 years	CATB	0.1076	0.7897	0.0227	0.1050
		(0.0965, 0.1230)	(0.7770, 0.8024)		
Between 5–10 years	CATB	0.1212	0.7458	0.0410	0.1664
		(0.1144, 0.1294)	(0.7357, 0.7558)		
Within 10 years	CATB	0.2233	0.7739	0.0583	0.2161
		(0.2115, 0.2369)	(0.7660, 0.7818)		

AUC values remained relatively stable across prediction horizons, ranging from approximately 0.75 to 0.79, with overlapping confidence intervals. For the 2-year prediction task, the model achieved a comparatively high AUC (0.789) despite a low AP, indicating divergence between ranking-based discrimination and precision-recall performance under low outcome prevalence.

Calibration metrics are reported in *Table 3*. Brier scores increased from 0.008 for the 2-year horizon to 0.058 for the 10-year horizon, while log loss increased from 0.048 to 0.216 over the same period.

Threshold-dependent classification metrics computed at prevalence-based thresholds are summarized in *Table 4*. F1 scores increased from 0.016 for the 2-year horizon to 0.242 for the 10-year horizon. Precision increased from 0.008 to 0.146 across the same timeframes, while recall remained high across all prediction windows, ranging from 0.71 to 1.00. Balanced accuracy increased from 0.57 for the 2-year horizon to 0.70 for the 10-year horizon.

**Table 4:** Optimal model scores computed at the prevalence threshold

Hypertension Risk	Best Model	F1 Score	Precision	Recall	Balanced Accuracy
Within 2 years	LGBM	0.0156	0.0079	0.9880	0.5711
Between 2–5 years	XGB	0.0332	0.0169	1.0000	0.5000
Within 5 years	CATB	0.0645	0.0334	0.9424	0.6413
Between 5–10 years	CATB	0.1506	0.0838	0.7407	0.6813
Within 10 years	CATB	0.2421	0.1459	0.7105	0.7029

### Explainability results (SHAP)

**Figure 3:**
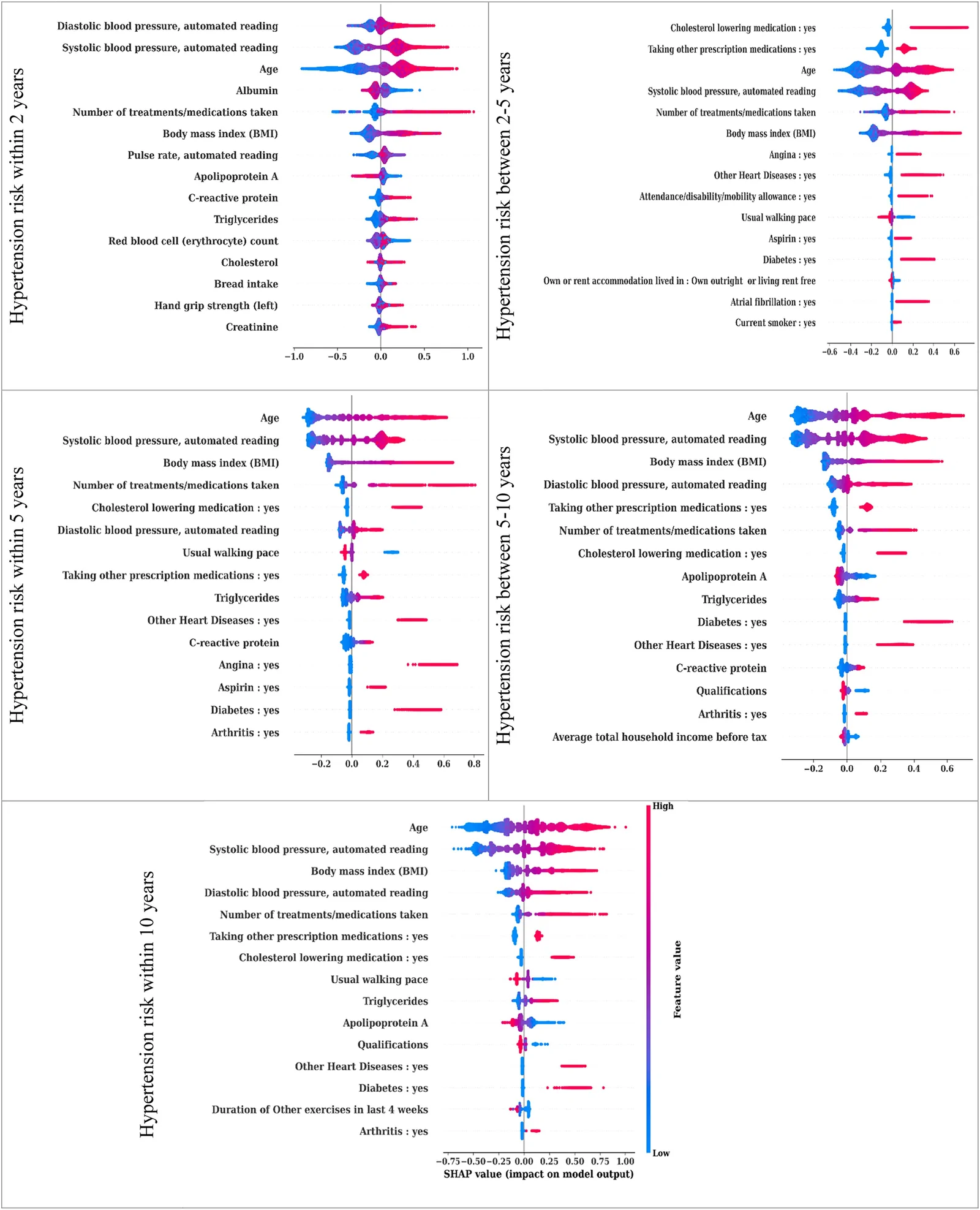
SHAP summary plots of feature importance across hypertension risk prediction horizons. Each panel shows the top 15 most influential features for the best-performing model at each prediction horizon. Points represent individual participants, coloured by feature magnitude (blue = low, red = high), with horizontal position indicating the SHAP value and the direction and strength of each feature’s contribution to predicted hypertension risk. Positive mean SHAP values (rightward) correspond to increased predicted risk, whereas negative values (leftward) indicate reduced predicted risk.

The SHAP summary plots in *Figure 3* present the top 15 influential features for each hypertension risk prediction task.

Across all prediction horizons, baseline automated systolic blood pressure measurements consistently emerged as one of the most influential predictors. Baseline diastolic blood pressure also demonstrated strong and stable importance, ranking among the top five features for longer prediction horizons (between 5–10 years and within 10 years) and among the most influential predictors for the shortest cumulative horizon (within 2 years).

Age exhibited a clear horizon-dependent pattern, with increasing importance observed for longer prediction windows. It emerged as the most influential feature for horizons of 5 years and beyond (within 5 years, between 5–10 years, and within 10 years), while remaining of moderate importance for shorter-term predictions. Body mass index (BMI) showed a similar pattern, ranking among the top three predictors for horizons of 5 years and beyond and among the top six for shorter-term intervals. Walking pace remained consistently influential across all horizons beyond 2 years except for the 5–10 years interval, with a higher walking pace associated with lower predicted hypertension risk.

Medication-related variables were also identified as important contributors, with their relative importance varying across prediction horizons. Cholesterol-lowering medication use ranked among the most influential features for intermediate-term prediction (between 2–5 years). Broader medication indicators, including ‘taking other prescription medications’ and ‘number of medications taken’, consistently appeared among the top-ranked features for longer-term prediction horizons.

Indicators of multimorbidity history displayed diverse patterns of importance across timeframes. Cardiovascular conditions and diabetes appeared consistently among the top predictors across multiple horizons. Heart disease indicators remained influential across all classification tasks beyond 2 years. Angina and atrial fibrillation showed greater prominence in the intermediate-term (2–5 years) prediction model, while arthritis appeared more prominently in longer horizons (i.e. within 5 years and beyond).

Blood-based biomarkers, including triglycerides, apolipoprotein A, and C-reactive protein, exhibited greater importance in longer-term CATB models and in the short-term cumulative LGBM model. In contrast, shorter segmented prediction windows (2–5 years) were characterized by greater prominence of clinical measurements, medication history, and multimorbidity indicators among the top-ranked features.

### Population-level visualization of predicted risk using GTM

**Figure 4:**
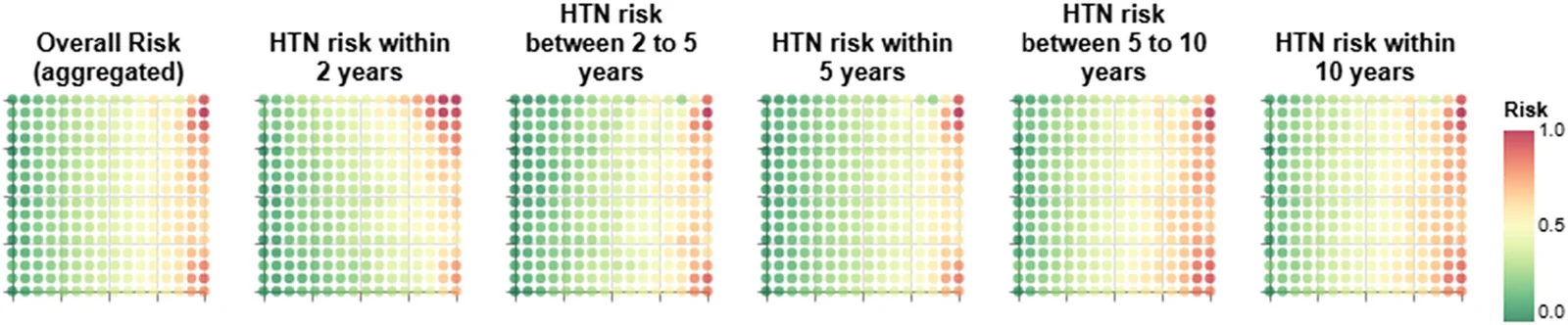
GTM reference maps of predicted hypertension risk across temporal horizons. Population-level visualization of prediction probability distributions for each hypertension risk horizon and an aggregated overall risk map. Each node represents a latent reference point in the learned topology, with colour indicating the predicted risk probability (green = low risk, red = high risk).

The GTM analysis revealed distinct topological patterns in the distribution of hypertension risk predictions and associated patient characteristics across the learnt prediction space. Reference maps corresponding to the five hypertension risk prediction outcomes demonstrated different clustering patterns across temporal horizons (*Figure 4*). Short-term risk predictions (within 2 years) exhibited high-intensity regions primarily located in the upper right areas of the topology, whereas longer-term risk predictions (between 5–10 years, within 10 years) displayed more distributed patterns, with higher intensities observed across central as well as the upper and lower regions in the right. The aggregated risk map captured these combined patterns, highlighting regions of overlapping high predicted risk across multiple time horizons.

**Figure 5:**
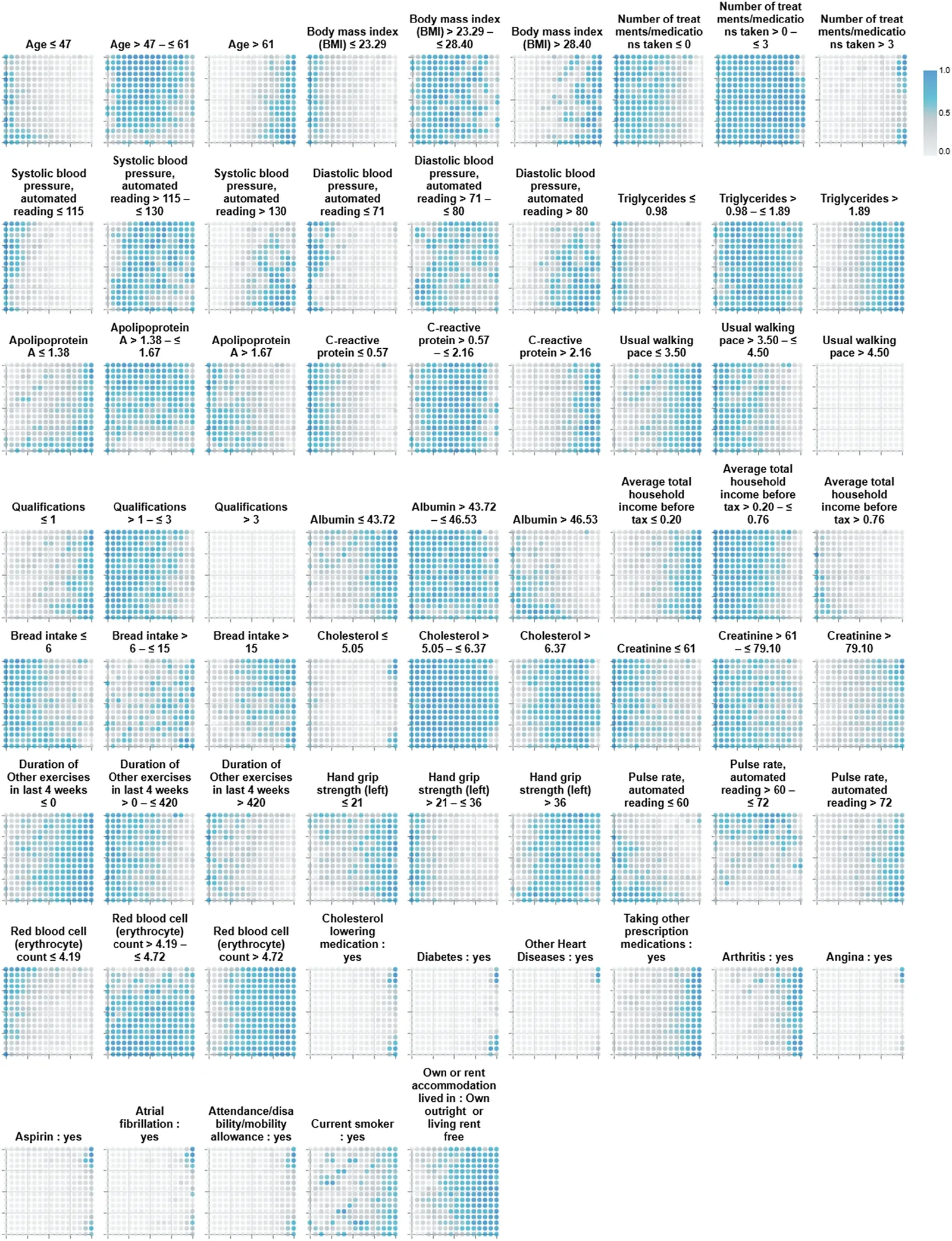
Superimposition of influential features on the GTM prediction space. Population-level visualizations showing the spatial distribution of discretized feature values over the learned GTM topology. Features were selected as the union of the top 15 contributors across all prediction horizons based on SHAP analysis and were not used in training the GTM. Colour intensity indicates the relative prevalence of each feature value within regions of the prediction space, illustrating how combinations of clinical, lifestyle, and socioeconomic factors align with different predicted hypertension risk patterns.

A total of 30 unique influential features were identified from the union of the top 15 contributing features across all classification tasks. Following the discretization of numerical variables into quartile-based bins and one-hot encoding, 68 investigative variables were superimposed onto the trained GTM (*Figure 5*). Age-related variables formed distinct spatial patterns, with younger age groups (≤47 years) predominantly occupying regions associated with lower predicted risk, and older age groups (>61 years) concentrated in regions associated with higher predicted risk.

Variables representing related physiological domains exhibited spatial proximity within the topology. Cardiovascular-related indicators, including blood pressure measurements and lipid-related variables, clustered within overlapping high-intensity regions of the prediction space. Elevated BMI, higher blood pressure values, and increased triglyceride levels were co-located within regions corresponding to higher predicted risk. Blood biomarkers, including cholesterol-related measures, C-reactive protein, and creatinine, displayed similar spatial organization, with higher values concentrated in regions of increased predicted risk. In contrast, apolipoprotein A and albumin levels showed an inverse spatial pattern, with higher values localized in regions associated with lower predicted risk.

Physical activity and lifestyle-related variables demonstrated clear spatial separation across the topology. A faster walking pace (>4.5) was predominantly localized to regions associated with lower predicted risk. Exercise duration variables also followed similar spatial distributions. Hand grip strength largely showed lower values concentrated in regions associated with higher predicted risk. Medication-related variables exhibited strong clustering patterns, with higher numbers of medications (>3) localized to regions of elevated predicted risk. Specific medication categories and multimorbidity indicators, including cholesterol-lowering medications, diabetes, heart disease, arthritis, and angina, were co-localized within similar high-risk regions of the topology.

Socioeconomic variables also demonstrated structured spatial patterns. Higher educational qualifications and household income were localized predominantly within regions associated with lower predicted risk, while lower socioeconomic indicators occupied regions corresponding to higher predicted risk. Housing-related variables followed notable spatial distributions, with ownership more prevalent in high-risk regions.

Distinct temporal patterns were observed across prediction horizons. Short-term risk (within 2 years) was primarily associated with regions characterized by elevated clinical measurements, higher medication burden, and existing comorbidities. Intermediate-term risk (2–5 years) showed increased co-localization with metabolic and lifestyle-related variables. Longer-term predictions (5–10 years) exhibited broader spatial associations encompassing lifestyle and socioeconomic variables. The cumulative 10-year risk category encompassed regions influenced by both clinical and longer-term lifestyle-related factors.

These GTM visualizations provide a population-level representation of how combinations of clinical, lifestyle, and socioeconomic variables are distributed across predicted hypertension risk profiles over multiple temporal horizons.

## Discussion

This study demonstrates that population-scale, multi-horizon prediction of incident hypertension is feasible and clinically informative even under extreme class imbalance, provided that evaluation strategies, model interpretation, and intended use cases are carefully aligned. Using UK Biobank data, we developed and evaluated multiple ML models across five predefined, clinically motivated time horizons. This framework extends prior studies (e.g. Zhao *et al*.^19^ Schjerven *et al*.^25^) that have predominantly relied on cross-sectional or single-horizon designs and delineates how discrimination, precision, and feature importance vary across temporal risk horizons.

Although our prediction models were assessed over time, we intentionally avoided survival modelling. Given the low prevalence of hypertension events in short-term windows, hazard-based approaches may have produced limited statistical power and unstable estimates, with an increased risk of overfitting.^30^ Instead, fixed horizon classification allowed consistent evaluation across the predefined time windows while retaining flexibility to accommodate extreme class imbalance. Within this framework, ensemble boosting methods demonstrated robust and consistent discrimination across horizons, but performance remained constrained by low positive predictive value in short-term windows, reflecting the statistical limitations imposed by rare-event prevalence.

From a clinical perspective, short-term prediction models, characterized by high sensitivity but low precision, may be more suitable for broad risk flagging or preliminary triage in population screening contexts. In contrast, longer-horizon models, with improved precision and more balanced classification performance, may better support targeted prevention strategies and multi-horizon risk monitoring.

Across all horizons, tree-based ensemble models (particularly boosting algorithms) outperformed traditional baseline classifiers (LR and NB). This observation is consistent with prior work showing that boosting methods perform well on complex, heterogeneous tabular data by capturing non-linear interactions and reducing bias and variance through iterative learning.^31^ However, differences among the leading boosting methods were small, suggesting that feature representation, evaluation strategy, computational efficiency, and deployability may be as important as the specific boosting implementation. In this regard, CATB demonstrated particularly strong and stable performance at longer prediction horizons, suggesting robustness as outcome prevalence (i.e. hypertension) increased and risk profiles became more heterogeneous over time. The variation in the best-performing algorithm across horizons may reflect differences in model architecture, with boosting approaches better able to capture non-linear interactions and weak horizon-specific signals under class imbalance; however, these differences should be interpreted cautiously because performance is also influenced by outcome prevalence, feature signal strength, and hyperparameter optimization.

While ensemble boosting models demonstrated consistently superior discrimination, their complexity introduces well-recognized challenges regarding interpretability.^11,21,32–34^ In clinical risk prediction, opacity may limit trust and implementation, particularly when models are used for preventive stratification rather than diagnostic support. To address this, we integrated complementary explainability approaches, combining feature-level attribution (SHAP),^35^ with topology-based population visualization (GTM).^36^ However, enhanced transparency should not be conflated with causal explanation. Accordingly, SHAP and GTM were used as tools for model transparency and support structured interpretation.

A central methodological contribution of this work lies in clarifying how evaluation metrics behave under extreme class imbalance and across varying prediction horizons. While ROC-AUC values remained relatively stable across time windows (0.75–0.79), average precision increased substantially with longer follow-up, tracking changes in hypertension prevalence. The divergence between high ROC-AUC and low average precision in short-term prediction highlights a critical limitation of relying solely on ROC-based metrics in low-prevalence settings. In clinical terms, a model may rank individuals correctly according to relative hypertension risk yet still generate a high proportion of false positives when identifying those predicted to develop the condition. Thus, strong rank discrimination does not necessarily translate into high positive predictive value. This reinforces current recommendations to prioritize precision-recall metrics when evaluating clinical risk models for rare outcomes,^37^ particularly when the intended use is not broad screening but risk stratification.

When contextualized within the existing literature, the discriminative performance achieved in this study is comparable to prior hypertension prediction efforts. For example, Kanegae *et al*.^24^ reported higher AUC values (0.88) using Japanese health check-up data, but their analysis relied on two years of prior annual health assessments to predict new-onset hypertension at the subsequent visit and was limited to a substantially smaller cohort of 18 258 subjects. Similarly, Fang *et al*.^20^ reported high AUC (0.95) and F1 scores (0.87) for five-year prediction, though their analysis relied on a near-balanced dataset (49% positive cases) from 33 255 records, a condition rarely encountered in real-world healthcare data. By preserving the natural prevalence of hypertension in UK Biobank (0.67–6.82%), our study reflects more realistic population-level conditions, resulting in constrained precision and lower F1 scores, particularly for short-term horizons; an expected trade-off in rare-event prediction.

Schjerven *et al*.^25^ reported relatively similar AUC (0.79) values for 11-year hypertension risk prediction using XGB in the Norwegian HUNT study (17 852 participants, 24% incidence), based on a reduced set of 18 features. To facilitate a more direct methodological comparison, we conducted additional exploratory analyses in which the UK Biobank feature set was restricted to a comparable reduced subset. These experiments yielded inferior performance relative to our full-feature models (see Supplementary Material  *S6*), despite aligning model class and outcome horizon, underscoring the importance of richer, multimodal feature representations for capturing long-term hypertension risk trajectories in large population cohorts.

Interpretability analyses provided important insight into the temporal structure of risk factors for hypertension development. Baseline systolic and diastolic blood pressure emerged as dominant predictors for future hypertension in the short-term horizons, indicating that proximity to diagnostic thresholds (i.e. elevated resting blood pressure) increases the likelihood of hypertension diagnosis, along with the presence of latent or undiagnosed hypertension in the baseline measurements.^38,39^ This finding should also be interpreted considering the known limitations of single-visit blood pressure measurements. Transient elevation due to factors such as white-coat effects or masked hypertension may contribute to baseline misclassification while still signalling increased near-term risk of subsequent hypertension diagnosis.^40^ In contrast, age and BMI became increasingly important over longer prediction horizons (i.e. ≤5y and beyond), supporting their established role as cumulative, long-term vascular and metabolic risk factors rather than immediate triggers for hypertension.^41,42^ Walking pace showed a consistent inverse association with hypertension risk, aligning with prior evidence linking it to reduced cardiometabolic risk.^43,44^

Multimorbidity indicators further contributed to the temporal differentiation of hypertension risk. Conditions such as diabetes and cardiovascular disease were consistently associated with increased risk across multiple horizons, in line with their well-established pathophysiological links to hypertension.^45^ Other conditions, including atrial fibrillation and angina, exhibited greater prominence in intermediate-term prediction windows, while arthritis appeared more relevant in longer horizons (i.e. ≤5 years and beyond).

Medication-related features at baseline also emerged as informative predictors, with cholesterol-lowering therapy and overall medication burden consistently associated with increased hypertension risk across multiple horizons. Rather than reflecting direct pharmacological effects, these variables likely function as proxies for underlying cardiometabolic risk, multimorbidity burden, and healthcare utilization patterns.^46^ In parallel, blood-based biomarkers (including triglycerides, apolipoprotein A, and C-reactive protein) gained prominence in longer-term and cumulative prediction horizons, aligning with their established roles in metabolic dysregulation, systemic inflammation, and progressive vascular risk accumulation.^47^

Socioeconomic factors gained prominence in longer-term predictions, with variables related to education, income, and housing conditions contributing more strongly to extended risk horizons (i.e. > 5 years). These observations are consistent with prior evidence linking socioeconomic disadvantage to higher hypertension prevalence and poorer cardiovascular outcomes.^48^

The integration of GTM alongside SHAP-based feature attribution provided complementary population-level insight into the structure of predicted hypertension risk. While SHAP enabled identification of influential features within individual models, GTM visualizations illustrated how combinations of clinical, lifestyle, and socioeconomic factors co-occurred across distinct regions of the prediction space and varied across temporal horizons. Used as an exploratory but topology-preserving approach, GTM enabled visualization of how risk-related features co-occurred across the prediction space.

The proposed framework has potential implications for real-world deployment in healthcare systems. For example, integration with existing risk assessment pathways, such as routine health checks or primary care screening programmes, could enable risk stratification across different time horizons. By identifying individuals at elevated medium- or long-term risk, such models may support earlier lifestyle interventions, monitoring, or targeted follow-up, potentially reducing the downstream burden of hypertension-related complications.

### Strengths

This study has several notable strengths. These include the use of a large, well-characterized prospective cohort; evaluation across five prediction horizons; performance assessment tailored to severe class imbalance; integration of SHAP and GTM for complementary interpretability; and inclusion of a broad multimodal feature set spanning sociodemographic, clinical, biomarker, lifestyle, medication, and multimorbidity domains.

### Limitations

Several limitations warrant consideration. While we employed a multi-horizon framework for risk prediction, the variables used were assessed only at baseline. As a result, their predictive contributions reflected static risk stratification rather than dynamic changes. Features such as medication use, biomarker, and age, are inherently dynamic and interdependent and are likely to change over time, thereby influencing hypertension risk. This limits interpretation of the models as dynamic risk predictors and motivates future analyses incorporating repeated measurements.

Extreme class imbalance constrained precision, particularly for short-term prediction windows, limiting the immediate utility of the models for individual-level risk estimation. While class weighting was employed to mitigate imbalance during model training, alternative strategies (including anomaly detection, representation learning, and generative modelling approaches) may offer additional performance gains and merit further investigation.^49–51^ In addition, although the inclusion of a large and diverse feature set enhanced predictive performance, reliance on more than 130 variables poses challenges for external validation and clinical implementation due to data availability and generalizability constraints.

Feature selection, therefore, represents an important direction for future work. Future work should explore clinically informed feature reduction strategies, provided these are coupled with rigorous validation and domain expertise. Causal inference frameworks may also help assess the modifiability and downstream impact of key risk factors, strengthening links between risk stratification, prevention, and intervention.^52^

In addition, the absence of external validation limits assessment of generalizability beyond the UK Biobank population and should be addressed in future studies using independent cohorts. Model performance may be influenced by cohort-specific demographic and clinical characteristics, including age distribution, ethnicity, and comorbidity burden. External validation in independent cohorts will therefore be necessary to assess transferability and clinical applicability.

## Methods

This study is reported in accordance with the TRIPOD (Transparent Reporting of a multivariable prediction model for Individual Prognosis or Diagnosis) checklist^53^ (see Supplementary material—*S6*).

### Data definition and screening

Data were extracted from the UK Biobank,^26^ for participants attending baseline assessment visits between 2006 and 2010. Only data from this initial assessment (Visit 1) were analysed.

Incident primary hypertension (International Classification of Diseases code: I10) was identified using the first recorded diagnosis date from hospital admissions, primary care records, self-report, or death registries. Independent variables comprised sociodemographic, lifestyle, psychosocial, medication, physical, cardiovascular, and blood biomarker measures.

Multimorbidity history included 36 chronic conditions defined by Chudasama *et al*.^54^ plus three additional conditions (heart disease, obesity, and visual impairment) previously associated with hypertension.^55–57^ Conditions were considered present if recorded on or before baseline. Hypertensive disorders indicative of pre-existing hypertension (e.g. hypertensive heart disease, hypertensive renal disease, secondary hypertension) were excluded to avoid circularity. Full variable definitions and derivations are provided in Supplementary Materials  *S7*-*S8*. Additional implementation details are available in the analysis code (see File  *S1*.1).

The cohort was defined according to the following criteria using relevant UK Biobank data fields:

1. Exclusion of participants diagnosed with hypertension prior to initial assessment.
2. Exclusion of participants diagnosed within 3 months post-baseline to reduce reverse causation.
3. Absence of hypertension at baseline (normotensive <140/90 mmHg, using data-fields: 4079, 4080).
4. Exclusion of individuals with prior antihypertensive treatment, i.e. participants reporting regular blood pressure medication use at baseline (data-fields: 6177, 6153). It should be noted that these fields do not specify prescription indication; therefore, medications prescribed for alternative indications cannot be distinguished.
5. Exclusion of individuals with hypertensive disorders (relevant data fields present in Supplementary Material  *S8*).
6. Exclusion of pregnant women (data-field: 3140).

Participants developing hypertension after the lag period were classified as incident cases; all others served as controls.

Risk groups were defined using fixed temporal intervals (≤2 years, 2–5 years, 5–10 years, >10 years) based on time from baseline to first diagnosis. Five binary outcome variables were constructed representing hypertension risk across different time horizons (i.e. ≤2 years, 2–5 years, ≤5 years, 5–10 years, ≤10 years) with cumulative categories incorporating cases from their constituent intervals. The 2-year window reflects near-term clinical risk, 2–5-year and 5-year horizons align with routine health assessment intervals and established risk scores^58^ and 5–10-year windows capture longer-term preventive risk relevant to primary prevention strategies.^2,59^ The maximum horizon was restricted to 10 years to ensure an adequate sample size and model stability.

**Figure 6:**
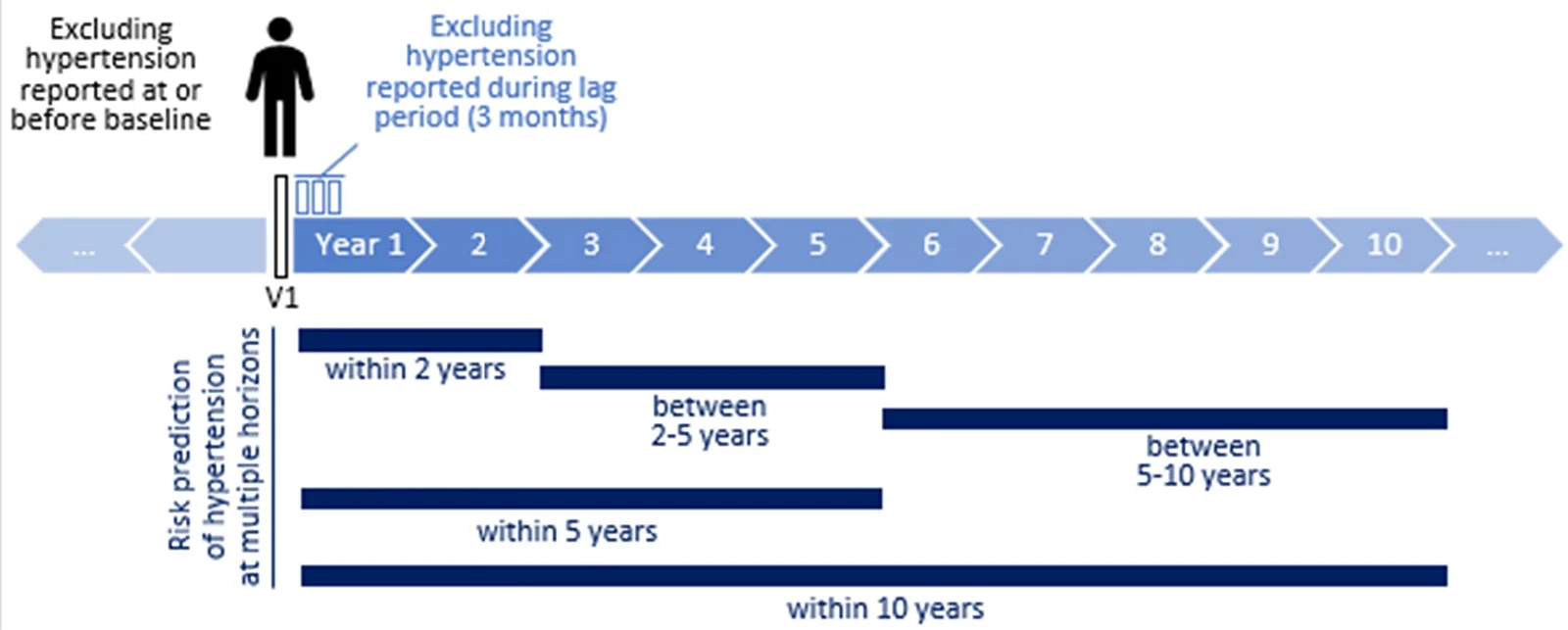
Cohort eligibility and outcome categorization overview. Primary exclusion criteria involved eliminating participants with HTN on or before V1, along with those diagnosed within 3 months after V1 (lag period). All remaining participants, following further exclusions, were included for incident hypertension prediction across multiple horizons.

Cohort selection and outcome definitions are illustrated in *Figure 6*. For each classification task, incident cases within the specified horizon were labelled positive, and all others negative (see Supplementary material online, Figure F1).

### Statistical analysis

The distribution of retrieved variables was assessed for normality using both statistical and visual approaches, using the Kolmogorov-Smirnov test along with histograms and quantile–quantile (Q–Q) plots,^60^ respectively. Data were examined for outliers using the interquartile range (IQR) method. Relationships among variables were explored by evaluating pairwise correlations between continuous variables and measures of association among categorical variables. Thresholds of 0.8 (correlation) and 0.5 (association) were used to denote strong relationships.^61^

Features exceeding these thresholds were reviewed, and the variable with the higher mean correlation or association across features was removed. Variables with more than 25% missing data were excluded, followed by the removal of participant records with more than 25% missing values.

The final dataset was stratified by hypertension risk group and summarized descriptively. Group comparisons were conducted using chi-square tests for categorical variables and Kruskal–Wallis or ANOVA for continuous variables, as appropriate,^62^ with Bonferroni correction applied for multiple testing.^63^

### Pre-processing

The final was partitioned into stratified training and test sets using an 80:20 split for each outcome variable, ensuring balanced class representation within each prediction horizon. All pre-processing steps were fitted on the training data and applied to the test set to prevent data leakage.

Missing values were imputed within age- and sex-specific strata using the median (continuous variables) and mode (categorical variables). More complex imputation methods were not implemented to preserve computational efficiency and reproducibility. Categorical features were encoded using one-hot encoding, and continuous variables were scaled using a Robust Scaler (median centring with interquartile range scaling) to reduce sensitivity to outliers.

### Modelling

A supervised ML framework was employed to model multiple binary classification tasks across distinct target variables using seven classification algorithms: Logistic Regression (LR),^64^ Naïve Bayes (NB),^65^ Random Forest (RF),^66^ Gradient Boosting (GB),^67^ Extreme Gradient Boosting (aka XGBoost or XGB),^68^ Categorical Boosting (aka CatBoost or CATB),^69^ and Light Gradient Boosting Machine (aka LightGBM or LGBM).^70^ All models were trained on the pre-processed data and evaluated independently for each target variable to ensure reproducibility and comparability. Hyperparameter optimization was conducted using Optuna,^71^ a Bayesian optimization framework, with model performance assessed via stratified 10-fold cross-validation. Average precision was used as the optimization objective due to its robustness under severe class imbalance and its ability to capture ranking performance. Optimal configurations were identified separately for each prediction horizon.

#### Model selection rationale

Model selection was guided by the objective of comparing baseline classifiers with advanced ensemble methods while balancing predictive performance and computational efficiency. All analyses were performed on the UK Biobank Research Analysis Platform, an Amazon Web Services–based cloud environment with usage-based compute costs. Given the scale of the dataset, computational efficiency was a key constraint, necessitating limits on hyperparameter optimization. Accordingly, the number of Optuna trials was constrained to 10–50 per model, depending on algorithm complexity, prioritizing configurations that provided favourable performance-resource trade-offs.

#### Baseline models

LR was employed as a baseline model due to its simplicity and interpretability. The SAGA solver was used with both L1 and L2 regularization to support implicit feature selection and mitigate overfitting. The regularization strength (C) was tuned on a logarithmic scale (0.01–1000), tolerance values ranged from 10^−4^ to 0.1, and class weighting was optimized between default and balanced settings.

NB was implemented as a hybrid model combining Gaussian Naive Bayes for continuous variables and Bernoulli Naive Bayes for binary variables, following the approach described by Godinez *et al*.^72^ Predictions from both components were averaged with equal weighting to produce final class probabilities. The variance smoothing parameter was tuned on a logarithmic scale (10^−15^ to 10^−1^), alongside Laplace smoothing (alpha: 0.1–2.0) to address zero probability issues.

#### Ensemble models

RF was implemented as a bagging ensemble of decision trees to capture non-linear relationships. Key hyperparameters included the number of estimators (100–500), maximum tree depth (5–30 or unrestricted), minimum samples for node splitting (8–48) and leaf nodes (4–24), and feature subsampling using the square root of the total number of features. Both balanced and balanced-subsample class weighting strategies were evaluated, with maximum sample fractions per tree ranging from 0.5 to 0.9 to enhance robustness while maintaining computational efficiency.

#### Boosting ensemble models

GB model was trained as an ensemble of sequentially optimized decision trees, where each tree attempts to correct the errors of its predecessors. To control computational cost, the number of estimators was limited to 100–300, with learning rates between 0.01 and 0.5. Model complexity was regulated via maximum tree depth (5–30 or unrestricted), minimum leaf samples (12–60), feature subsampling strategies, and subsampling ratios (0.6–0.85).

XGB was evaluated for its efficiency, regularization capabilities, and support for parallel and GPU-accelerated training. Both L1 and L2 regularization terms (0–7) were tuned, alongside tree-specific parameters including maximum depth (5–50 or unrestricted), minimum child weight (1–20), gamma (0–1), and independent row and column subsampling ratios (0.5–0.95 and 0.4–0.95, respectively). Class imbalance correction was addressed through optimization of the positive class weight, including both the raw class ratio and its square root.

CATB was assessed for its robustness to overfitting through symmetric tree construction and ordered boosting. Models were trained using CPU-based execution with Bayesian bootstrapping, with depths ranging from 4 to 8, learning rates between 0.01 and 0.2, and 100–500 boosting iterations. Regularization was controlled via L2 leaf regularization (1–7) and random strength (0.1–3). Class imbalance was addressed using both manually specified class weights (1:2, 1:3, 1:5) and automatically computed balanced weights.

LGBM was employed for its computational efficiency and scalability, using histogram-based tree construction. The number of estimators ranged from 100 to 500, with key hyperparameters including the number of leaves (31–255), minimum child samples (20–100), and maximum bin size (15–255). Multiple boosting strategies (‘gbdt’, ‘rf’, and ‘dart’) were evaluated, alongside L1 and L2 regularization terms (0–3). As with XGB, the positive class weight was optimized using both the raw imbalance ratio and its square root.

All optimal models for each algorithm and outcome variable, identified via hyperparameter optimization and cross-validation, were subsequently evaluated using consistent performance metrics appropriate for imbalanced binary classification, as described in the following section.

### Performance evaluation

The selection of appropriate performance metrics is critical under severe class imbalance. In accordance with recommended practices,^73^ model performance was primarily assessed using average precision (AP) and the area under the receiver operating characteristic curve (AUC). AP was prioritized because it reflects precision-recall performance under rare-event conditions, while AUC was used as a complementary measure of discrimination.

The Brier score and log loss were computed to assess probabilistic calibration. The Brier score quantifies the mean squared error of predicted probabilities, whereas log loss evaluates the negative log-likelihood. Together, these metrics assess the reliability of predicted risks.

Additional threshold-dependent metrics (F1 score, balanced accuracy, precision, and recall) were calculated at a decision threshold defined by class prevalence. This ensured consistent evaluation across horizons without *post hoc* threshold optimization. Finally, 95% CIs for AP and AUC were estimated to quantify statistical uncertainty.

### Model explainability using SHAP

SHapley Additive exPlanations (SHAP), a widely used explainable AI technique, was employed to interpret feature contributions.^35^ SHAP values, grounded in cooperative game theory,^74^ quantify the average marginal contribution of each feature to a model’s predictions across all possible feature subsets. This enables both global interpretability (via aggregated feature importance) and local interpretability at the individual prediction level.

Although classical SHAP is model-agnostic, its exponential computational complexity limits scalability for large datasets and complex tree-based models. Accordingly, TreeSHAP^75^ was used for all tree-based ensembles, enabling efficient computation of exact SHAP values in polynomial time by exploiting tree structure and dynamic programming. For each prediction task, TreeSHAP was applied to the best-performing model to identify and rank the top 15 contributing features, providing a consistent and computationally efficient assessment of global feature importance across prediction horizons.

### GTM-based framework for population-level risk visualization

While SHAP identifies influential features within individual models, it does not capture how combinations of features organize predicted risk across the population. To complement feature-level attribution, GTM,^36,76^ a probabilistic manifold learning approach, was applied *post hoc* to explore the structure of model predictions and associated feature patterns.

GTM models a non-linear mapping from a low-dimensional latent space to the observed data space and assigns observations to latent components via posterior probabilities, enabling topology-preserving visualization under uncertainty.^77^ Unlike hard clustering methods, GTM provides soft assignments, allowing overlapping risk structures to be represented on a continuous membership map.

Prediction probabilities from the five optimal binary classifiers (corresponding to the predefined hypertension horizons) were used as input features, forming a five-dimensional probability space. GTM was trained on this space to identify latent reference vectors (cluster centres), which were visualized as reference maps (heatmaps) representing composite prediction patterns.

Utilizing the methods set out in Bellfield *et al*.^78^ the union of the top 15 SHAP-ranked features across models was defined as a set of investigative variables. Numerical variables were discretized into quartiles and one-hot encoded. These variables were not included during GTM training but were projected onto the learned topology to examine how feature distributions varied across regions of predicted risk.

An overall risk representation was additionally derived by aggregating the five reference vectors, yielding a composite topology of hypertension risk across multiple time horizons.

## Conclusion

Population-scale, multi-horizon prediction of incident hypertension is achievable using machine learning when supported by appropriate evaluation, interpretability, and visualization strategies. Ensemble boosting methods demonstrated robust performance across clinically meaningful time horizons, with improved utility for medium- and long-term risk stratification under severe class imbalance. The integration of complementary explainability approaches (SHAP and GTM) enabled both feature-level interpretation and population-level characterization of risk structures. By capturing how hypertension risk evolves across time, this framework provides a foundation for more targeted, time-specific prevention strategies and supports the potential integration of AI-driven risk stratification into routine cardiovascular care.

## Supplementary Material

ztag128_Supplementary_Data

## Data Availability

The UK Biobank database is available for approved projects only (application process detailed at https://www.ukbiobank.ac.uk/enable-your-research/apply-for-access) through the UK Biobank Access Management System (https://www.ukbiobank.ac.uk).

## Code availability

The code is available here: DOI: 10.5281/zenodo.21904587.
