# Supplementary material for: Multi-horizon machine learning for population-level hypertension risk stratification in the UK Biobank
Source: Eur Heart J Digit Health. 2026 Aug 10;7(7):ztag128. doi: 10.1093/ehjdh/ztag128 (PMC13505867; doi:10.1093/ehjdh/ztag128)
Supplement: Supplementary file 1 [file ztag128_supplementary_data.zip › Supplementary Material S1 - List of Supplementary Figures.docx]

**List of Supplementary Figures**

**F 1. Definition of binary outcome variables across hypertension risk horizons.** Schematic illustrating how each dependent variable was derived based on the timing of first reported hypertension. Green indicates assignment to the positive class (1), and red indicates the negative class (0) for each prediction task.

**F 2. Distribution of time to incident hypertension following the baseline assessment.** Histogram and density plot showing the number of years from the first assessment visit to the first recorded diagnosis of hypertension among incident cases. Vertical dashed lines indicate the median and interquartile range, with additional summary statistics shown in the inset.

**F 3. Timeline of participants’ assessment visits.** Distribution of participant attendance across the initial and subsequent UK Biobank assessment visits by calendar year, showing fewer participants attending later follow-up visits.
