# Supplementary material for: Multi-horizon machine learning for population-level hypertension risk stratification in the UK Biobank
Source: Eur Heart J Digit Health. 2026 Aug 10;7(7):ztag128. doi: 10.1093/ehjdh/ztag128 (PMC13505867; doi:10.1093/ehjdh/ztag128)
Supplement: Supplementary file 1 [file ztag128_supplementary_data.zip › Supplementary Material S2 - Variables eliminated.docx]

Table 1. Variables eliminated due to significant correlation

| Correlation | Variable retained | Variable eliminated* |
| --- | --- | --- |
| 0.93 | Apolipoprotein A | HDL cholesterol |
| 0.91 | Hand grip strength (left) | Hand grip strength (right) |
| 0.88 | Direct bilirubin | Total bilirubin |
| 0.87 | Cholesterol | Apolipoprotein B |
| 0.86 | Alcohol intake frequency | Alcohol average weekly intake |
| 0.86 | Tobacco smoking status | Pack years of smoking |
| 0.81 | Red blood cell (erythrocyte) count | Haemoglobin concentration |
| 0.81 | Body mass index (BMI) | Weight |
| *Note: Variables eliminated based on higher overall mean correlation | | |
