# Supplementary material for: Multi-horizon machine learning for population-level hypertension risk stratification in the UK Biobank
Source: Eur Heart J Digit Health. 2026 Aug 10;7(7):ztag128. doi: 10.1093/ehjdh/ztag128 (PMC13505867; doi:10.1093/ehjdh/ztag128)
Supplement: Supplementary file 1 [file ztag128_supplementary_data.zip › Supplementary Material S3 - Statistical Description.pdf]

Statistical Measures :-	**Categorical :** n - Count, % - Percent/Proportion,<br>**Ordinal :** Median, Q1 - First Quartile, Q3 - Third Quartile,<br>**Continuous :** Mean, SD - Standard Deviation		Grouped by Hypertension Risk								
**Category**	**Variables**		**Missing**	**Overall**	**within 2 years**	**etween 2-5 yea**	**etween 5-10 years**	**after 10 years**	**no htn**	**P-Value (adjusted)**	**Test**
	n			246286	1661	4154	10988	10117	219366		
Vairable used to derive dependant/target variables	Hypertension Risk, n (%)	after 10 years	0	10117 (4.1078)	0 (0.0000)	0 (0.0000)	0 (0.0000)	10117 (100.0000)	0 (0.0000)	<0.001	Chi-squared
		between 2-5 years		4154 (1.6867)	0 (0.0000)	4154 (100.0000)	0 (0.0000)	0 (0.0000)	0 (0.0000)		
		between 5-10 years		10988 (4.4615)	0 (0.0000)	0 (0.0000)	10988 (100.0000)	0 (0.0000)	0 (0.0000)		
		no htn		219366 (89.0696)	0 (0.0000)	0 (0.0000)	0 (0.0000)	0 (0.0000)	219366 (100.0000)		
		within 2 years		1661 (0.6744)	1661 (100.0000)	0 (0.0000)	0 (0.0000)	0 (0.0000)	0 (0.0000)		
Personal Features	Sex, n (%)	Female	0	149904 (60.8658)	821 (49.4281)	2132 (51.3240)	5840 (53.1489)	5633 (55.6786)	135478 (61.7589)	<0.001	Chi-squared
		Male		96382 (39.1342)	840 (50.5719)	2022 (48.6760)	5148 (46.8511)	4484 (44.3214)	83888 (38.2411)		
	Age, median [Q1,Q3]		0	54.0000 [47.0000,61.0000]	60.0000 [54.0000,65.0000]	60.0000 [53.0000,65.0000]	59.0000 [52.0000,64.0000]	58.0000 [51.0000,63.0000]	54.0000 [47.0000,60.0000]	<0.001	Kruskal-Wallis
Sociodemographic Features	Type of accommodation lived in, n (%)	None of the above	191	528 (0.2146)	4 (0.2418)	14 (0.3375)	37 (0.3370)	22 (0.2176)	451 (0.2057)	<0.001	Chi-squared (warning: expected count < 5)
		Permanent Housing		244913 (99.5197)	1637 (98.9722)	4099 (98.8187)	10889 (99.1712)	10054 (99.4461)	218234 (99.5579)		
		Specialized or Supported Housing		389 (0.1581)	12 (0.7255)	28 (0.6750)	40 (0.3643)	23 (0.2275)	286 (0.1305)		
		Temporary or Mobile Housing		265 (0.1077)	1 (0.0605)	7 (0.1688)	14 (0.1275)	11 (0.1088)	232 (0.1058)		
	Own or rent accommodation lived in, n (%)	None of the above	1835	1694 (0.6930)	21 (1.2766)	46 (1.1157)	99 (0.9087)	74 (0.7371)	1454 (0.6677)	<0.001	Chi-squared
		Own outright or living rent free		114925 (47.0135)	889 (54.0426)	2176 (52.7771)	5861 (53.7953)	5349 (53.2822)	100650 (46.2229)		
		Own with a mortgage Or Paying Rent		127041 (51.9699)	725 (44.0729)	1887 (45.7676)	4896 (44.9380)	4589 (45.7117)	114944 (52.7874)		
		Pay part rent and part mortgage (shared ownership)		791 (0.3236)	10 (0.6079)	14 (0.3396)	39 (0.3580)	27 (0.2690)	701 (0.3219)		
	Number in household, median [Q1,Q3]		1069	2.0000 [2.0000,3.0000]	2.0000 [2.0000,3.0000]	2.0000 [2.0000,3.0000]	2.0000 [2.0000,3.0000]	2.0000 [2.0000,3.0000]	2.0000 [2.0000,3.0000]	<0.001	Kruskal-Wallis
	Average total household income before tax, mean (SD)		31310	0.4261 (0.2873)	0.3242 (0.2607)	0.3331 (0.2670)	0.3426 (0.2674)	0.3691 (0.2746)	0.4351 (0.2879)	<0.001	One-way ANOVA
	Current employment status, n (%)	Employed	928	163887 (66.7951)	746 (45.1574)	2048 (49.5284)	5677 (51.9016)	5667 (56.2481)	149749 (68.5168)	<0.001	Chi-squared
		Not Employed		81471 (33.2049)	906 (54.8426)	2087 (50.4716)	5261 (48.0984)	4408 (43.7519)	68809 (31.4832)		
	Qualifications, median [Q1,Q3]		2045	3.0000 [1.0000,3.0000]	2.0000 [1.0000,3.0000]	2.0000 [1.0000,3.0000]	2.0000 [1.0000,3.0000]	2.0000 [1.0000,3.0000]	3.0000 [1.0000,3.0000]	<0.001	Kruskal-Wallis
	Ethnic background, n (%)	Asian or Asian British	810	9892 (4.0297)	47 (2.8416)	131 (3.1612)	357 (3.2644)	359 (3.5580)	8998 (4.1152)	<0.001	Chi-squared
		Black or Black British		1492 (0.6078)	12 (0.7255)	33 (0.7963)	88 (0.8047)	78 (0.7730)	1281 (0.5859)		
		Chinese		915 (0.3727)	4 (0.2418)	13 (0.3137)	20 (0.1829)	22 (0.2180)	856 (0.3915)		
		Mixed		9283 (3.7816)	71 (4.2926)	199 (4.8021)	481 (4.3983)	384 (3.8057)	8148 (3.7265)		
		Other ethnic group		2388 (0.9728)	16 (0.9674)	58 (1.3996)	112 (1.0241)	85 (0.8424)	2117 (0.9682)		
		White		221506 (90.2353)	1504 (90.9311)	3710 (89.5270)	9878 (90.3255)	9162 (90.8028)	197252 (90.2128)		
	Attendance/disability/mobility allowance, n (%)	no	1568	235066 (96.0559)	1412 (86.0451)	3640 (88.3924)	9910 (91.0344)	9379 (93.5468)	210725 (96.6420)	<0.001	Chi-squared
		yes		9652 (3.9441)	229 (13.9549)	478 (11.6076)	976 (8.9656)	647 (6.4532)	7322 (3.3580)		
Family History	Parents suffering from high blood pressure, n (%)	both	0	15830 (6.4275)	102 (6.1409)	281 (6.7646)	704 (6.4070)	681 (6.7312)	14062 (6.4103)	<0.001	Chi-squared
		father		28919 (11.7420)	167 (10.0542)	428 (10.3033)	1161 (10.5661)	1144 (11.3077)	26019 (11.8610)		
		mother		48416 (19.6584)	306 (18.4226)	811 (19.5234)	2214 (20.1493)	1984 (19.6106)	43101 (19.6480)		
		none		131248 (53.2909)	866 (52.1373)	2127 (51.2037)	5699 (51.8657)	5265 (52.0411)	117291 (53.4682)		
		unknown		21873 (8.8811)	220 (13.2450)	507 (12.2051)	1210 (11.0120)	1043 (10.3094)	18893 (8.6125)		
Medication	Number of treatments/medications taken, median [Q1,Q3]		165	1.0000 [0.0000,3.0000]	3.0000 [1.0000,5.0000]	2.0000 [1.0000,4.0000]	2.0000 [1.0000,4.0000]	2.0000 [0.0000,3.0000]	1.0000 [0.0000,2.0000]	<0.001	Kruskal-Wallis
	Taking other prescription medications, n (%)	no	641	148090 (60.2862)	587 (35.4897)	1568 (37.8927)	4576 (41.7709)	4825 (47.8196)	136534 (62.3990)	<0.001	Chi-squared
		yes		97555 (39.7138)	1067 (64.5103)	2570 (62.1073)	6379 (58.2291)	5265 (52.1804)	82274 (37.6010)		
	Vitamin and mineral supplements, n (%)	no	745	164239 (66.8886)	1118 (67.7576)	2828 (68.3422)	7247 (66.2674)	6598 (65.5148)	146448 (66.9489)	0.530	Chi-squared
		yes		81302 (33.1114)	532 (32.2424)	1310 (31.6578)	3689 (33.7326)	3473 (34.4852)	72298 (33.0511)		
	Mineral and other dietary supplements, n (%)	no	412	142746 (58.0566)	916 (55.2806)	2342 (56.5291)	6060 (55.2768)	5474 (54.1819)	127954 (58.4243)	<0.001	Chi-squared
		yes		103128 (41.9434)	741 (44.7194)	1801 (43.4709)	4903 (44.7232)	4629 (45.8181)	91054 (41.5757)		
	Cholesterol lowering medication, n (%)	no	0	228900 (92.9407)	1187 (71.4630)	3217 (77.4434)	9003 (81.9348)	8770 (86.6858)	206723 (94.2366)	<0.001	Chi-squared
		yes		17386 (7.0593)	474 (28.5370)	937 (22.5566)	1985 (18.0652)	1347 (13.3142)	12643 (5.7634)		
	Insulin, n (%)	no	0	245135 (99.5327)	1620 (97.5316)	4074 (98.0741)	10831 (98.5712)	10012 (98.9621)	218598 (99.6499)	<0.001	Chi-squared
		yes		1151 (0.4673)	41 (2.4684)	80 (1.9259)	157 (1.4288)	105 (1.0379)	768 (0.3501)		
	Aspirin, n (%)	no	2190	226761 (92.8983)	1248 (75.9586)	3336 (81.4254)	9304 (85.7274)	8860 (88.6177)	204013 (93.7969)	<0.001	Chi-squared
		yes		17335 (7.1017)	395 (24.0414)	761 (18.5746)	1549 (14.2726)	1138 (11.3823)	13492 (6.2031)		
	Ibuprofen (e.g. Nurofen), n (%)	no	2190	202765 (83.0677)	1411 (85.8795)	3449 (84.1835)	9098 (83.8294)	8337 (83.3867)	180470 (82.9728)	0.084	Chi-squared

	Ibuprofen (e.g. Nurofen), n (%)	yes	2190	41331 (16.9323)	232 (14.1205)	648 (15.8165)	1755 (16.1706)	1661 (16.6133)	37035 (17.0272)	0.084	Chi-squared
	Paracetamol, n (%)	no	2190	189350 (77.5719)	1197 (72.8545)	2947 (71.9307)	7997 (73.6847)	7510 (75.1150)	169699 (78.0207)	<0.001	Chi-squared
		yes		54746 (22.4281)	446 (27.1455)	1150 (28.0693)	2856 (26.3153)	2488 (24.8850)	47806 (21.9793)		
	Ranitidine (e.g. Zantac), n (%)	no	2190	240156 (98.3859)	1591 (96.8351)	3967 (96.8269)	10559 (97.2911)	9762 (97.6395)	214277 (98.5159)	<0.001	Chi-squared
		yes		3940 (1.6141)	52 (3.1649)	130 (3.1731)	294 (2.7089)	236 (2.3605)	3228 (1.4841)		
	Omeprazole (e.g. Zanprol), n (%)	no	2190	233461 (95.6431)	1459 (88.8010)	3655 (89.2116)	9915 (91.3572)	9264 (92.6585)	209168 (96.1670)	<0.001	Chi-squared
		yes		10635 (4.3569)	184 (11.1990)	442 (10.7884)	938 (8.6428)	734 (7.3415)	8337 (3.8330)		
	Laxatives (e.g. Dulcolax, Senokot), n (%)	no	2190	236955 (97.0745)	1556 (94.7048)	3896 (95.0940)	10355 (95.4114)	9628 (96.2993)	211520 (97.2483)	<0.001	Chi-squared
		yes		7141 (2.9255)	87 (5.2952)	201 (4.9060)	498 (4.5886)	370 (3.7007)	5985 (2.7517)		
Psychosocial Factors	Frequency of friend/family visits, mean (SD)		1325	0.2919 (0.2887)	0.3250 (0.3080)	0.3245 (0.3162)	0.3230 (0.3086)	0.3154 (0.3032)	0.2884 (0.2861)	<0.001	One-way ANOVA
	Leisure/social activities, n (%)	no	733	72902 (29.6889)	499 (30.1511)	1350 (32.6639)	3483 (31.8344)	3075 (30.5029)	64495 (29.4844)	<0.001	Chi-squared
		yes		172651 (70.3111)	1156 (69.8489)	2783 (67.3361)	7458 (68.1656)	7006 (69.4971)	154248 (70.5156)		
	Able to confide, mean (SD)		7353	0.5978 (0.4452)	0.5536 (0.4580)	0.5631 (0.4570)	0.5646 (0.4540)	0.5717 (0.4502)	0.6016 (0.4441)	<0.001	One-way ANOVA
	Neuroticism score, median [Q1,Q3]		46510	4.0000 [1.0000,6.0000]	4.0000 [1.0000,7.0000]	4.0000 [2.0000,7.0000]	4.0000 [2.0000,7.0000]	4.0000 [2.0000,7.0000]	4.0000 [1.0000,6.0000]	<0.001	Kruskal-Wallis
	Risk taking, n (%)	no	9385	171223 (72.2762)	1148 (71.4819)	2834 (71.3854)	7442 (70.6139)	6886 (70.6184)	152913 (72.4586)	<0.001	Chi-squared
		yes		65678 (27.7238)	458 (28.5181)	1136 (28.6146)	3097 (29.3861)	2865 (29.3816)	58122 (27.5414)		
	Frequency of depressed mood in last 2 weeks, mean (SD)		10788	0.1093 (0.2121)	0.1331 (0.2435)	0.1398 (0.2446)	0.1245 (0.2321)	0.1177 (0.2239)	0.1074 (0.2095)	<0.001	One-way ANOVA
	Frequency of unenthusiasm / disinterest in last 2 weeks, mean (SD)		8567	0.0978 (0.2086)	0.1303 (0.2434)	0.1271 (0.2355)	0.1151 (0.2278)	0.1077 (0.2167)	0.0957 (0.2063)	<0.001	One-way ANOVA
	Frequency of tenseness / restlessness in last 2 week, mean (SD)		9359	0.1174 (0.2122)	0.1423 (0.2395)	0.1376 (0.2352)	0.1308 (0.2283)	0.1242 (0.2207)	0.1159 (0.2102)	<0.001	One-way ANOVA
	Frequency of tiredness / lethargy in last 2 weeks, mean (SD)		6920	0.2489 (0.2782)	0.2969 (0.3134)	0.2937 (0.3023)	0.2853 (0.2985)	0.2691 (0.2928)	0.2449 (0.2754)	<0.001	One-way ANOVA
	Illness, injury, bereavement, stress in last 2 years, n (%)	no	1321	134358 (54.8478)	836 (50.8825)	2135 (52.0605)	5625 (51.5299)	5295 (52.6028)	120467 (55.1996)	<0.001	Chi-squared
		yes		110607 (45.1522)	807 (49.1175)	1966 (47.9395)	5291 (48.4701)	4771 (47.3972)	97772 (44.8004)		
	Seen doctor (GP) or psychiatrist for nerves, anxiety, tension or depression, n (%)	no	407	159329 (64.7998)	988 (59.5899)	2488 (60.0676)	6507 (59.3325)	6181 (61.2041)	143165 (65.3683)	<0.001	Chi-squared
		yes		86550 (35.2002)	670 (40.4101)	1654 (39.9324)	4460 (40.6675)	3918 (38.7959)	75848 (34.6317)		
Environmental Factors	Time spend outdoors in summer, mean (SD)		13084	3.5759 (2.3199)	4.0026 (2.4937)	3.9524 (2.5321)	3.8980 (2.4345)	3.7902 (2.3513)	3.5399 (2.3044)	<0.001	One-way ANOVA
	Time spend outdoors in winter, mean (SD)		12815	1.8191 (1.7269)	2.0746 (1.8974)	2.0813 (1.9661)	2.0365 (1.8957)	1.9777 (1.8219)	1.7943 (1.7058)	<0.001	One-way ANOVA
	Exposure to tobacco smoke, median [Q1,Q3]		38871	0.0000 [0.0000,0.0000]	0.0000 [0.0000,1.0000]	0.0000 [0.0000,1.0000]	0.0000 [0.0000,0.0000]	0.0000 [0.0000,0.0000]	0.0000 [0.0000,0.0000]	<0.001	Kruskal-Wallis
Smoking	Current smoker, n (%)	no	726	217538 (88.5885)	1404 (85.0394)	3464 (83.9147)	9255 (84.6442)	8647 (85.8433)	194768 (89.0270)	<0.001	Chi-squared
		yes		28022 (11.4115)	247 (14.9606)	664 (16.0853)	1679 (15.3558)	1426 (14.1567)	24006 (10.9730)		
	Tobacco smoking status, median [min,max]		129	0.0000 [0.0000,3.0000]	0.0000 [0.0000,3.0000]	0.0000 [0.0000,3.0000]	0.0000 [0.0000,3.0000]	0.0000 [0.0000,3.0000]	0.0000 [0.0000,3.0000]	<0.001	Kruskal-Wallis
Alcohol	Alcohol drinker status, n (%)	Current	227	227502 (92.4583)	1460 (88.1643)	3666 (88.4866)	9865 (89.9271)	9212 (91.2079)	203299 (92.7501)	<0.001	Chi-squared
		Never		10260 (4.1697)	105 (6.3406)	253 (6.1067)	584 (5.3236)	473 (4.6832)	8845 (4.0353)		
		Previous		8297 (3.3720)	91 (5.4952)	224 (5.4067)	521 (4.7493)	415 (4.1089)	7046 (3.2146)		
	Alcohol intake frequency, mean (SD)		168	0.3704 (0.3466)	0.3383 (0.3536)	0.3417 (0.3537)	0.3522 (0.3548)	0.3667 (0.3557)	0.3723 (0.3455)	<0.001	One-way ANOVA
Sleep Features	Sleep duration, median [Q1,Q3]		1159	7.0000 [7.0000,8.0000]	7.0000 [6.0000,8.0000]	7.0000 [6.0000,8.0000]	7.0000 [6.0000,8.0000]	7.0000 [6.0000,8.0000]	7.0000 [7.0000,8.0000]	<0.001	Kruskal-Wallis
	Sleeplessness / insomnia, median [Q1,Q3]		149	2.0000 [1.0000,3.0000]	2.0000 [2.0000,3.0000]	2.0000 [2.0000,3.0000]	2.0000 [2.0000,3.0000]	2.0000 [2.0000,3.0000]	2.0000 [1.0000,3.0000]	<0.001	Kruskal-Wallis
Physical Activity	Usual walking pace, mean (SD)		124	3.8148 (0.8273)	3.4768 (0.9669)	3.5400 (0.9359)	3.6086 (0.9043)	3.6959 (0.8552)	3.8384 (0.8151)	<0.001	One-way ANOVA
	Frequency of stair climbing in last 4 weeks, median [Q1,Q3]		1694	8.0000 [3.0000,13.0000]	8.0000 [3.0000,13.0000]	8.0000 [3.0000,13.0000]	8.0000 [3.0000,13.0000]	8.0000 [3.0000,13.0000]	8.0000 [3.0000,13.0000]	<0.001	Kruskal-Wallis
	Duration of walks per week, median [Q1,Q3]		2792	200.0000 [90.0000,420.0000]	180.0000 [90.0000,360.0000]	200.0000 [90.0000,395.0000]	180.0000 [90.0000,420.0000]	200.0000 [90.0000,420.0000]	200.0000 [90.0000,420.0000]	0.039	Kruskal-Wallis
	Duration of moderate activity per week, median [Q1,Q3]		10114	120.0000 [40.0000,270.0000]	120.0000 [30.0000,280.0000]	120.0000 [30.0000,300.0000]	120.0000 [30.0000,270.0000]	120.0000 [40.0000,300.0000]	120.0000 [40.0000,270.0000]	1.000	Kruskal-Wallis
	Duration of vigorous activity per week, median [Q1,Q3]		9660	30.0000 [0.0000,120.0000]	20.0000 [0.0000,90.0000]	15.0000 [0.0000,90.000	20.0000 [0.0000,90.0000]	20.0000 [0.0000,90.0000]	30.0000 [0.0000,120.0000]	<0.001	Kruskal-Wallis
	Duration of sedentary activity per week, median [Q1,Q3]		0	1680.0000 [1260.0000,2520.0000]	2100.0000 [1470.0000,2730.0000]	2100.0000 [1470.0000,2520.0000]	1890.0000 [1470.0000,2520.0000]	1890.0000 [1260.0000,2520.0000]	1680.0000 [1260.0000,2310.0000]	<0.001	Kruskal-Wallis
	Duration of Heavy DIY in last 4 weeks, mean (SD)		8350	188.3355 (578.7013)	170.9686 (548.8014)	196.6306 (659.5664)	194.1698 (614.4579)	207.5468 (623.9672)	187.1398 (573.2688)	0.636	One-way ANOVA

	Duration of Light DIY in last 4 weeks, mean (SD)		5418	293.7931 (700.5730)	265.3182 (693.6172)	289.2469 (758.8884)	293.7994 (726.8554)	299.1047 (729.7996)	293.8474 (696.7815)	1.000	One-way ANOVA
	Duration of Other exercises in last 4 weeks, mean (SD)		2024	303.2144 (539.4445)	243.6300 (537.9035)	235.9156 (504.8319)	249.7680 (526.0873)	273.8253 (543.8082)	308.9378 (540.2422)	<0.001	One-way ANOVA
	Duration of Strenuous sports in last 4 weeks, mean (SD)		1022	86.6953 (328.6722)	47.5396 (250.6508)	45.3093 (223.5947)	52.4663 (264.9735)	64.3237 (293.6205)	90.5087 (334.9818)	<0.001	One-way ANOVA
	Duration of Walking for pleasure in last 4 weeks, mean (SD)		1880	424.6095 (656.2868)	406.0901 (700.6751)	408.9088 (665.3742)	421.8059 (701.1275)	425.4937 (684.3797)	425.1427 (652.1250)	1.000	One-way ANOVA
Diet	Cooked vegetable intake, mean (SD)		2612	2.6714 (1.9056)	2.7779 (2.4655)	2.7733 (2.2345)	2.7353 (2.0252)	2.7640 (2.0089)	2.6612 (1.8826)	<0.001	One-way ANOVA
	Salad / raw vegetable intake, mean (SD)		2593	2.2191 (2.1733)	2.1253 (2.9525)	2.1624 (2.3044)	2.1773 (2.2450)	2.2392 (2.2185)	2.2221 (2.1580)	1.000	One-way ANOVA
	Fresh fruit intake, mean (SD)		826	2.2243 (1.5761)	2.2868 (2.0383)	2.2178 (1.6109)	2.2081 (1.7064)	2.2315 (1.6044)	2.2245 (1.5634)	1.000	One-way ANOVA
	Dried fruit intake, mean (SD)		2366	0.8714 (1.7404)	0.8679 (1.8402)	0.7826 (1.6016)	0.8553 (2.1703)	0.8537 (1.7275)	0.8747 (1.7184)	1.000	One-way ANOVA
	Bread intake, mean (SD)		2315	11.7629 (8.4792)	12.6194 (8.6015)	12.0266 (8.5505)	12.1995 (8.4628)	12.0975 (8.6498)	11.7143 (8.4684)	<0.001	One-way ANOVA
	Cereal intake, mean (SD)		742	4.5634 (2.7901)	4.3046 (2.8131)	4.4752 (2.8650)	4.4874 (2.8348)	4.5806 (2.7641)	4.5700 (2.7872)	0.002	One-way ANOVA
	Tea intake, mean (SD)		442	3.3955 (2.8577)	3.5290 (3.0945)	3.6340 (3.2068)	3.5579 (3.0462)	3.5474 (2.9694)	3.3749 (2.8330)	<0.001	One-way ANOVA
	Coffee intake, mean (SD)		454	2.0195 (2.0858)	2.1539 (2.4565)	2.0460 (2.1170)	2.1401 (2.3707)	2.1230 (2.1912)	2.0072 (2.0614)	<0.001	One-way ANOVA
	Water intake, mean (SD)		1492	2.7716 (2.2872)	2.7605 (2.3790)	2.7099 (2.2829)	2.7016 (2.3964)	2.6700 (2.3405)	2.7810 (2.2783)	<0.001	One-way ANOVA
	Oily fish intake, mean (SD)		1299	0.1515 (0.1462)	0.1631 (0.1611)	0.1548 (0.1532)	0.1541 (0.1501)	0.1568 (0.1509)	0.1510 (0.1456)	<0.001	One-way ANOVA
	Non-oily fish intake, mean (SD)		1117	0.1599 (0.1339)	0.1664 (0.1375)	0.1595 (0.1351)	0.1608 (0.1353)	0.1621 (0.1360)	0.1597 (0.1336)	1.000	One-way ANOVA
	Processed meat intake, mean (SD)		473	0.2002 (0.1967)	0.2267 (0.2067)	0.2155 (0.2026)	0.2147 (0.2036)	0.2107 (0.2008)	0.1985 (0.1959)	<0.001	One-way ANOVA
	Poultry intake, mean (SD)		371	0.2716 (0.1821)	0.2728 (0.1816)	0.2733 (0.1804)	0.2728 (0.1828)	0.2734 (0.1822)	0.2714 (0.1821)	1.000	One-way ANOVA
	Beef intake, mean (SD)		873	0.1222 (0.1199)	0.1337 (0.1308)	0.1335 (0.1271)	0.1292 (0.1260)	0.1248 (0.1192)	0.1214 (0.1194)	<0.001	One-way ANOVA
	Lamb/mutton intake, mean (SD)		1364	0.0814 (0.0766)	0.0865 (0.0905)	0.0886 (0.0832)	0.0873 (0.0822)	0.0875 (0.0813)	0.0806 (0.0758)	<0.001	One-way ANOVA
	Pork intake, mean (SD)		1291	0.0832 (0.0814)	0.0929 (0.0982)	0.0884 (0.0893)	0.0897 (0.0892)	0.0870 (0.0827)	0.0826 (0.0806)	<0.001	One-way ANOVA
	Cheese intake, mean (SD)		5975	0.3604 (0.2549)	0.3345 (0.2463)	0.3293 (0.2468)	0.3387 (0.2501)	0.3428 (0.2517)	0.3631 (0.2554)	<0.001	One-way ANOVA
	Salt added to food, median [Q1,Q3]		17	1.0000 [1.0000,2.0000]	2.0000 [1.0000,2.0000]	1.0000 [1.0000,2.0000]	2.0000 [1.0000,2.0000]	1.0000 [1.0000,2.0000]	1.0000 [1.0000,2.0000]	<0.001	Kruskal-Wallis
	Milk type used, n (%)	High fat dairy	8212	17693 (7.4317)	125 (7.8174)	296 (7.3760)	761 (7.1677)	679 (6.9321)	15832 (7.4662)	<0.001	Chi-squared
		Low fat dairy		205861 (86.4693)	1401 (87.6173)	3520 (87.7149)	9296 (87.5577)	8594 (87.7386)	183050 (86.3240)		
		Other		3396 (1.4264)	29 (1.8136)	65 (1.6197)	170 (1.6012)	125 (1.2762)	3007 (1.4181)		
		Soya		11124 (4.6725)	44 (2.7517)	132 (3.2893)	390 (3.6734)	397 (4.0531)	10161 (4.7918)		
	Coffee type, n (%)	Contains Caffeine	55985	153522 (80.6732)	1034 (81.1617)	2471 (79.4789)	6672 (79.5517)	6213 (79.2474)	137132 (80.8128)	0.025	Chi-squared
		Decaffeinated		36779 (19.3268)	240 (18.8383)	638 (20.5211)	1715 (20.4483)	1627 (20.7526)	32559 (19.1872)		
	Cereal type, n (%)	Refined & Processed Cereals	42236	38502 (18.8689)	313 (23.5515)	769 (22.7380)	1888 (21.0081)	1784 (21.2685)	33748 (18.5465)	<0.001	Chi-squared
		Whole Grain & High-Fiber Cereals		165548 (81.1311)	1016 (76.4485)	2613 (77.2620)	7099 (78.9919)	6604 (78.7315)	148216 (81.4535)		
	Bread type, n (%)	Refined flour or other bread	10273	67573 (28.6310)	538 (33.6460)	1383 (34.3517)	3492 (33.0150)	3029 (31.1914)	59131 (28.1442)	<0.001	Chi-squared
		Whole Grain & Less Processed Bread		168440 (71.3690)	1061 (66.3540)	2643 (65.6483)	7085 (66.9850)	6682 (68.8086)	150969 (71.8558)		
	Spread type, n (%)	Butter	366	93988 (38.2189)	634 (38.2850)	1511 (36.4448)	4045 (36.9002)	3730 (36.9307)	84068 (38.3774)	<0.001	Chi-squared
		Never/rarely use spread		28815 (11.7172)	162 (9.7826)	445 (10.7332)	1143 (10.4269)	1143 (11.3168)	25922 (11.8335)		
		Non Butter		123117 (50.0638)	860 (51.9324)	2190 (52.8220)	5774 (52.6729)	5227 (51.7525)	109066 (49.7891)		
Cardiovascular Assessment	Diastolic blood pressure, automated reading, median [Q1,Q3]		19121	75.0000 [70.0000,81.0000]	78.0000 [72.0000,83.0000]	78.0000 [72.0000,83.0000]	78.0000 [73.0000,83.0000]	78.0000 [72.0000,82.0000]	75.0000 [70.0000,80.0000]	<0.001	Kruskal-Wallis
	Systolic blood pressure, automated reading, median [Q1,Q3]		19123	123.0000 [115.0000,130.0000]	127.0000 [120.0000,134.0000]	128.0000 [121.0000,134.0000]	128.0000 [121.0000,134.0000]	127.0000 [120.0000,133.0000]	122.0000 [114.0000,130.0000]	<0.001	Kruskal-Wallis
	Pulse rate, automated reading, median [Q1,Q3]		19121	66.0000 [60.0000,73.0000]	68.0000 [61.0000,76.0000]	68.0000 [61.0000,75.0000]	67.0000 [61.0000,75.0000]	67.0000 [60.0000,74.0000]	66.0000 [60.0000,72.0000]	<0.001	Kruskal-Wallis
Physcial Measures	Hand grip strength (left), median [Q1,Q3]		982	28.0000 [21.0000,36.0000]	28.0000 [20.0000,37.0000]	28.0000 [20.0000,37.0000]	28.0000 [20.0000,36.0000]	28.0000 [20.0000,37.0000]	28.0000 [21.0000,36.0000]	<0.001	Kruskal-Wallis
	Standing height, mean (SD)		590	168.3536 (9.1768)	168.3925 (9.2979)	168.2670 (9.5055)	168.3385 (9.3909)	168.4601 (9.3411)	168.3508 (9.1511)	1.000	One-way ANOVA
	Body mass index (BMI), mean (SD)		781	26.1814 (4.2157)	28.4438 (4.9913)	28.3080 (5.0318)	27.8721 (4.7443)	27.3533 (4.4733)	25.9859 (4.1072)	<0.001	One-way ANOVA
Blood [illegible]	Red blood cell (erythrocyte) count, mean (SD)		9665	4.4595 (0.4069)	4.5028 (0.4406)	4.5144 (0.4169)	4.5069 (0.4163)	4.4913 (0.4151)	4.4543 (0.4053)	<0.001	One-way ANOVA
	Albumin, mean (SD)		33784	45.1330 (2.5849)	44.5181 (2.6634)	44.6180 (2.6477)	44.7152 (2.6156)	44.7699 (2.5500)	45.1850 (2.5782)	<0.001	One-way ANOVA
	Apolipoprotein A, mean (SD)		34931	1.5504 (0.2682)	1.4836 (0.2609)	1.4799 (0.2670)	1.4965 (0.2634)	1.5162 (0.2670)	1.5565 (0.2679)	<0.001	One-way ANOVA
	Direct bilirubin, mean (SD)		51262	1.8078 (0.8222)	1.8702 (0.8440)	1.7962 (0.8166)	1.7916 (0.8400)	1.7857 (0.8490)	1.8093 (0.8201)	0.187	One-way ANOVA
	Cholesterol, mean (SD)		13639	5.7468 (1.0731)	5.6023 (1.2437)	5.6340 (1.2036)	5.7175 (1.1646)	5.7768 (1.1200)	5.7501 (1.0618)	<0.001	One-way ANOVA

Biomarkers	Creatinine, mean (SD)		13745	70.3513 (13.8947)	73.2554 (17.1826)	72.9640 (15.4697)	72.0501 (18.0817)	71.5612 (14.8790)	70.1394 (13.5266)	<0.001	One-way ANOVA
	C-reactive protein, mean (SD)		14109	2.2140 (4.0389)	3.0900 (4.3528)	3.3967 (5.6142)	2.9905 (4.9286)	2.6481 (4.2615)	2.1262 (3.9298)	<0.001	One-way ANOVA
	Lipoprotein A, mean (SD)		58006	44.2204 (48.7836)	46.4972 (50.5754)	45.5048 (49.4862)	45.5192 (49.4245)	45.3243 (48.9250)	44.0642 (48.7168)	0.328	One-way ANOVA
	Triglycerides, mean (SD)		13797	1.5933 (0.9439)	1.9011 (1.0983)	1.9060 (1.0693)	1.8678 (1.0973)	1.7896 (1.0283)	1.5623 (0.9227)	<0.001	One-way ANOVA
	Vitamin D, mean (SD)		23438	49.3367 (21.2378)	47.3215 (21.9150)	47.0553 (21.2272)	48.2863 (21.1755)	49.9768 (21.0412)	49.4173 (21.2403)	<0.001	One-way ANOVA
Multimorbidity History	Asthma, n (%)	no	0	217724 (88.4029)	1404 (84.5274)	3520 (84.7376)	9436 (85.8755)	8753 (86.5177)	194611 (88.7152)	<0.001	Chi-squared
		yes		28562 (11.5971)	257 (15.4726)	634 (15.2624)	1552 (14.1245)	1364 (13.4823)	24755 (11.2848)		
	Depression, n (%)	no	0	224030 (90.9634)	1453 (87.4774)	3621 (87.1690)	9645 (87.7776)	9014 (89.0976)	200297 (91.3072)	<0.001	Chi-squared
		yes		22256 (9.0366)	208 (12.5226)	533 (12.8310)	1343 (12.2224)	1103 (10.9024)	19069 (8.6928)		
	Diabetes, n (%)	no	0	241638 (98.1128)	1504 (90.5479)	3824 (92.0558)	10312 (93.8478)	9673 (95.6113)	216325 (98.6137)	<0.001	Chi-squared
		yes		4648 (1.8872)	157 (9.4521)	330 (7.9442)	676 (6.1522)	444 (4.3887)	3041 (1.3863)		
	Angina, n (%)	no	0	243109 (98.7100)	1485 (89.4040)	3850 (92.6818)	10482 (95.3950)	9826 (97.1237)	217466 (99.1339)	<0.001	Chi-squared
		yes		3177 (1.2900)	176 (10.5960)	304 (7.3182)	506 (4.6050)	291 (2.8763)	1900 (0.8661)		
	Migraine, n (%)	no	0	234447 (95.1930)	1558 (93.7989)	3947 (95.0169)	10401 (94.6578)	9606 (94.9491)	208935 (95.2449)	0.276	Chi-squared
		yes		11839 (4.8070)	103 (6.2011)	207 (4.9831)	587 (5.3422)	511 (5.0509)	10431 (4.7551)		
	Eczema or dermatitis, n (%)	no	0	220087 (89.3624)	1464 (88.1397)	3671 (88.3727)	9746 (88.6968)	9052 (89.4732)	196154 (89.4186)	1.000	Chi-squared
		yes		26199 (10.6376)	197 (11.8603)	483 (11.6273)	1242 (11.3032)	1065 (10.5268)	23212 (10.5814)		
	Irritable bowel syndrome, n (%)	no	0	232549 (94.4223)	1534 (92.3540)	3839 (92.4169)	10250 (93.2836)	9495 (93.8519)	207431 (94.5593)	<0.001	Chi-squared
		yes		13737 (5.5777)	127 (7.6460)	315 (7.5831)	738 (6.7164)	622 (6.1481)	11935 (5.4407)		
	Myocardial Infarction, n (%)	no	0	244714 (99.3617)	1543 (92.8958)	3963 (95.4020)	10738 (97.7248)	9970 (98.5470)	218500 (99.6052)	<0.001	Chi-squared
		yes		1572 (0.6383)	118 (7.1042)	191 (4.5980)	250 (2.2752)	147 (1.4530)	866 (0.3948)		
	Osteoporosis, n (%)	no	0	241174 (97.9244)	1599 (96.2673)	3989 (96.0279)	10659 (97.0058)	9825 (97.1138)	215102 (98.0562)	<0.001	Chi-squared
		yes		5112 (2.0756)	62 (3.7327)	165 (3.9721)	329 (2.9942)	292 (2.8862)	4264 (1.9438)		
	Stroke, n (%)	no	0	244549 (99.2947)	1616 (97.2908)	4058 (97.6890)	10830 (98.5621)	10007 (98.9127)	218038 (99.3946)	<0.001	Chi-squared
		yes		1737 (0.7053)	45 (2.7092)	96 (2.3110)	158 (1.4379)	110 (1.0873)	1328 (0.6054)		
	Anxiety or panic attacks, n (%)	no	0	232430 (94.3740)	1538 (92.5948)	3852 (92.7299)	10241 (93.2017)	9450 (93.4071)	207349 (94.5219)	<0.001	Chi-squared
		yes		13856 (5.6260)	123 (7.4052)	302 (7.2701)	747 (6.7983)	667 (6.5929)	12017 (5.4781)		
	Arthritis, n (%)	no	0	211379 (85.8266)	1181 (71.1017)	3064 (73.7602)	8365 (76.1285)	8044 (79.5097)	190725 (86.9437)	<0.001	Chi-squared
		yes		34907 (14.1734)	480 (28.8983)	1090 (26.2398)	2623 (23.8715)	2073 (20.4903)	28641 (13.0563)		
	Glaucoma, n (%)	no	0	243491 (98.8651)	1629 (98.0734)	4052 (97.5445)	10774 (98.0524)	9952 (98.3691)	217084 (98.9597)	<0.001	Chi-squared
		yes		2795 (1.1349)	32 (1.9266)	102 (2.4555)	214 (1.9476)	165 (1.6309)	2282 (1.0403)		
	Epilepsy, n (%)	no	0	243772 (98.9792)	1626 (97.8928)	4087 (98.3871)	10839 (98.6440)	9998 (98.8238)	217222 (99.0226)	<0.001	Chi-squared
		yes		2514 (1.0208)	35 (2.1072)	67 (1.6129)	149 (1.3560)	119 (1.1762)	2144 (0.9774)		
	Vestibular disorder, n (%)	no	0	243691 (98.9463)	1635 (98.4347)	4096 (98.6038)	10820 (98.4711)	10003 (98.8732)	217137 (98.9839)	<0.001	Chi-squared
		yes		2595 (1.0537)	26 (1.5653)	58 (1.3962)	168 (1.5289)	114 (1.1268)	2229 (1.0161)		
	Atrial fibrillation, n (%)	no	0	244059 (99.0958)	1581 (95.1836)	4007 (96.4612)	10703 (97.4063)	9944 (98.2900)	217824 (99.2971)	<0.001	Chi-squared
		yes		2227 (0.9042)	80 (4.8164)	147 (3.5388)	285 (2.5937)	173 (1.7100)	1542 (0.7029)		
	Chronic sinusitis, n (%)	no	0	241746 (98.1566)	1618 (97.4112)	4050 (97.4964)	10720 (97.5610)	9886 (97.7167)	215472 (98.2249)	<0.001	Chi-squared
		yes		4540 (1.8434)	43 (2.5888)	104 (2.5036)	268 (2.4390)	231 (2.2833)	3894 (1.7751)		
	Tuberculosis, n (%)	no	0	245009 (99.4815)	1639 (98.6755)	4130 (99.4222)	10911 (99.2992)	10047 (99.3081)	218282 (99.5058)	<0.001	Chi-squared
		yes		1277 (0.5185)	22 (1.3245)	24 (0.5778)	77 (0.7008)	70 (0.6919)	1084 (0.4942)		
	Meningitis, n (%)	no	0	244975 (99.4677)	1648 (99.2173)	4124 (99.2778)	10915 (99.3356)	10055 (99.3872)	218233 (99.4835)	1.000	Chi-squared
		yes		1311 (0.5323)	13 (0.7827)	30 (0.7222)	73 (0.6644)	62 (0.6128)	1133 (0.5165)		
	Chronic Obstructive Pulmonary Disease (COPD), n (%)	no	0	242920 (98.6333)	1577 (94.9428)	4006 (96.4372)	10679 (97.1878)	9891 (97.7661)	216767 (98.8152)	<0.001	Chi-squared
		yes		3366 (1.3667)	84 (5.0572)	148 (3.5628)	309 (2.8122)	226 (2.2339)	2599 (1.1848)		
	Multiple Sclerosis, n (%)	no	0	245188 (99.5542)	1652 (99.4582)	4130 (99.4222)	10930 (99.4722)	10077 (99.6046)	218399 (99.5592)	1.000	Chi-squared
		yes		1098 (0.4458)	9 (0.5418)	24 (0.5778)	58 (0.5278)	40 (0.3954)	967 (0.4408)		
	Thyroid problem, n (%)	no	0	231096 (93.8324)	1515 (91.2101)	3799 (91.4540)	10080 (91.7364)	9377 (92.6856)	206325 (94.0551)	<0.001	Chi-squared
		yes		15190 (6.1676)	146 (8.7899)	355 (8.5460)	908 (8.2636)	740 (7.3144)	13041 (5.9449)		
	Parkinson's disease, n (%)	no	0	245870 (99.8311)	1652 (99.4582)	4138 (99.6148)	10959 (99.7361)	10097 (99.8023)	219024 (99.8441)	<0.001	Chi-squared (warning: expected count < 5)
		yes		416 (0.1689)	9 (0.5418)	16 (0.3852)	29 (0.2639)	20 (0.1977)	342 (0.1559)		
	Prostate problem, n (%)	no	0	240763 (97.7575)	1571 (94.5816)	3953 (95.1613)	10508 (95.6316)	9773 (96.5998)	214958 (97.9906)	<0.001	Chi-squared
		yes		5523 (2.2425)	90 (5.4184)	201 (4.8387)	480 (4.3684)	344 (3.4002)	4408 (2.0094)		
	Bronchiectasis, n (%)	no	0	245543 (99.6983)	1656 (99.6990)	4142 (99.7111)	10926 (99.4357)	10078 (99.6145)	218741 (99.7151)	<0.001	Chi-squared
		yes		743 (0.3017)	5 (0.3010)	12 (0.2889)	62 (0.5643)	39 (0.3855)	625 (0.2849)		
	Anaemia, n (%)	no	0	235974 (95.8130)	1554 (93.5581)	3940 (94.8483)	10483 (95.4041)	9720 (96.0759)	210277 (95.8567)	<0.001	Chi-squared

	**Anaemia, n (%)**	**yes**	0	10312 (4.1870)	107 (6.4419)	214 (5.1517)	505 (4.5959)	397 (3.9241)	9089 (4.1433)	<0.001	Chi-squared
	**Peripheral vascular disease, n (%)**	**no**	0	244154 (99.1343)	1625 (97.8326)	4080 (98.2186)	10832 (98.5803)	10006 (98.9028)	217611 (99.2000)	<0.001	Chi-squared
		**yes**		2132 (0.8657)	36 (2.1674)	74 (1.7814)	156 (1.4197)	111 (1.0972)	1755 (0.8000)		
	**Heart failure, n (%)**	**no**	0	245940 (99.8595)	1637 (98.5551)	4118 (99.1334)	10933 (99.4995)	10091 (99.7430)	219161 (99.9065)	<0.001	Chi-squared (warning: expected count < 5)
		**yes**		346 (0.1405)	24 (1.4449)	36 (0.8666)	55 (0.5005)	26 (0.2570)	205 (0.0935)		
	**Cirrhosis, n (%)**	**no**	0	246065 (99.9103)	1655 (99.6388)	4143 (99.7352)	10970 (99.8362)	10105 (99.8814)	219192 (99.9207)	<0.001	Chi-squared (warning: expected count < 5)
		**yes**		221 (0.0897)	6 (0.3612)	11 (0.2648)	18 (0.1638)	12 (0.1186)	174 (0.0793)		
	**Hepatitis, n (%)**	**no**	0	244020 (99.0799)	1646 (99.0969)	4100 (98.7000)	10885 (99.0626)	10005 (98.8930)	217384 (99.0965)	1.000	Chi-squared
		**yes**		2266 (0.9201)	15 (0.9031)	54 (1.3000)	103 (0.9374)	112 (1.1070)	1982 (0.9035)		
	**Schizophrenia, n (%)**	**no**	0	245823 (99.8120)	1646 (99.0969)	4133 (99.4945)	10952 (99.6724)	10091 (99.7430)	219001 (99.8336)	<0.001	Chi-squared (warning: expected count < 5)
		**yes**		463 (0.1880)	15 (0.9031)	21 (0.5055)	36 (0.3276)	26 (0.2570)	365 (0.1664)		
	**Dementia, n (%)**	**no**	0	246216 (99.9716)	1659 (99.8796)	4151 (99.9278)	10984 (99.9636)	10113 (99.9605)	219309 (99.9740)	1.000	Chi-squared (warning: expected count < 5)
		**yes**		70 (0.0284)	2 (0.1204)	3 (0.0722)	4 (0.0364)	4 (0.0395)	57 (0.0260)		
	**Chronic kidney disease, n (%)**	**no**	0	245269 (99.5871)	1635 (98.4347)	4101 (98.7241)	10887 (99.0808)	10061 (99.4465)	218585 (99.6440)	<0.001	Chi-squared
		**yes**		1017 (0.4129)	26 (1.5653)	53 (1.2759)	101 (0.9192)	56 (0.5535)	781 (0.3560)		
	**Inflammatory bowel disease, n (%)**	**no**	0	243427 (98.8392)	1628 (98.0132)	4083 (98.2908)	10817 (98.4438)	9966 (98.5075)	216933 (98.8909)	<0.001	Chi-squared
		**yes**		2859 (1.1608)	33 (1.9868)	71 (1.7092)	171 (1.5562)	151 (1.4925)	2433 (1.1091)		
	**Obesity, n (%)**	**no**	0	242492 (98.4595)	1591 (95.7857)	3997 (96.2205)	10698 (97.3608)	9872 (97.5783)	216334 (98.6178)	<0.001	Chi-squared
		**yes**		3794 (1.5405)	70 (4.2143)	157 (3.7795)	290 (2.6392)	245 (2.4217)	3032 (1.3822)		
	**Visual disturbances and blindness, n (%)**	**no**	0	243817 (98.9975)	1633 (98.3143)	4076 (98.1223)	10812 (98.3983)	9993 (98.7743)	217303 (99.0596)	<0.001	Chi-squared
		**yes**		2469 (1.0025)	28 (1.6857)	78 (1.8777)	176 (1.6017)	124 (1.2257)	2063 (0.9404)		
	**Other Heart Diseases, n (%)**	**no**	0	238113 (96.6815)	1413 (85.0692)	3690 (88.8300)	10121 (92.1096)	9519 (94.0892)	213370 (97.2667)	<0.001	Chi-squared
		**yes**		8173 (3.3185)	248 (14.9308)	464 (11.1700)	867 (7.8904)	598 (5.9108)	5996 (2.7333)		
	**Cancer, n (%)**	**no**	0	225162 (91.4230)	1469 (88.4407)	3706 (89.2152)	9791 (89.1063)	9146 (90.4023)	201050 (91.6505)	<0.001	Chi-squared
		**yes**		21124 (8.5770)	192 (11.5593)	448 (10.7848)	1197 (10.8937)	971 (9.5977)	18316 (8.3495)		
