# Supplementary material for: Multi-horizon machine learning for population-level hypertension risk stratification in the UK Biobank
Source: Eur Heart J Digit Health. 2026 Aug 10;7(7):ztag128. doi: 10.1093/ehjdh/ztag128 (PMC13505867; doi:10.1093/ehjdh/ztag128)
Supplement: Supplementary file 1 [file ztag128_supplementary_data.zip › Supplementary Material S4 - Optimal Hyperparameters.docx]

Table 1. Optimal Hyperparameters for best model within each prediction task

| Prediction Task | Best Model | Optimal Hyperparameters |
| --- | --- | --- |
| HTN risk within 2 years | LGBM | {'n_estimators': 300, 'max_depth': 15, 'num_leaves': 63, 'min_child_samples': 50, 'subsample': 0.9, 'colsample_bytree': 0.5, 'reg_alpha': 3.0, 'reg_lambda': 0.5, 'boosting_type': 'dart', 'subsample_freq': 1, 'max_bin': 15, 'scale_pos_weight': 5, 'tree_learner': 'feature_parallel'} |
| HTN risk between 2 to 5 years | XGB | {'n_estimators': 500, 'colsample_bytree': 0.9, 'subsample': 0.8, 'max_depth': None, 'learning_rate': 0.005, 'scale_pos_weight': 5, 'min_child_weight': 1, 'gamma': 1, 'reg_lambda': 5, 'reg_alpha': 0.1} |
| HTN risk within 5 years | CATB | {'depth': 6, 'learning_rate': 0.009999999776482582, 'iterations': 500, 'l2_leaf_reg': 7, 'class_weights': [1, 2], 'random_strength': 0.5} |
| HTN risk between 5 to 10 years | CATB | {'depth': 8, 'learning_rate': 0.009999999776482582, 'iterations': 400, 'l2_leaf_reg': 5, 'class_weights': [1, 1], 'random_strength': 0.10000000149011612} |
| HTN risk within 10 years* | CATB | {'depth': 4, 'learning_rate': 0.10000000149011612, 'iterations': 300, 'l2_leaf_reg': 1, 'class_weights': [1, 1], 'random_strength': 2} |
|  | | |
