# Supplementary material for: Multi-horizon machine learning for population-level hypertension risk stratification in the UK Biobank
Source: Eur Heart J Digit Health. 2026 Aug 10;7(7):ztag128. doi: 10.1093/ehjdh/ztag128 (PMC13505867; doi:10.1093/ehjdh/ztag128)
Supplement: Supplementary file 1 [file ztag128_supplementary_data.zip › Supplementary Material S5 - HTN prediction, limited vars.docx]

**Hypertension risk prediction with limited variables**

This analysis was performed to evaluate whether a restricted feature set, mirroring that employed in Schjerven et al.'s study on the HUNT dataset [1], would yield comparable predictive performance on UK Biobank data. The HUNT study employed 18 baseline features for 11-year hypertension incidence modeling via machine learning approaches like XGBoost and elastic net regression. Exact variable mappings to UK Biobank field IDs were not fully attainable, yet replication proceeded by selecting the closest phenotypic proxies to approximate this parsimonious set, as detailed in analysis code file 1.3. We used the below-mentioned variables to predict Hypertension risk at different temporal window i.e. within 2 years, between 2-5 years, within 5 years, between 5-10 years and within 10 years to align with our comparative analysis.

**List of Independent Variables Used**

| - Age - Sex - Standing height - Body mass index (BMI) - Systolic blood pressure, automated reading - Diastolic blood pressure, automated reading - Cholesterol - Creatinine | - Qualifications - Family Hypertension History - Physical Activity - Family CVD History - HDL cholesterol - Glucose - Smoking status - Married or Cohabiting - eGFR Stage - Triglycerides |
| --- | --- |

**Results :** Optimal model scores for each classification problem

| Hypertension Risk | Best Model | AP  (95% C.I) | AUC  (95% C.I) | Brier Score | Log Loss |
| --- | --- | --- | --- | --- | --- |
| Within 2 years | XGB | 0.0219 | 0.7531 | 0.0069 | 0.0434 |
|  |  | (0.0185, 0.0279) | (0.7284, 0.7779) |  |  |
| Between 2 to 5 years | CATB | 0.0529 | 0.7525 | 0.0167 | 0.0833 |
|  |  | (0.0477, 0.611) | (0.7366, 0.7684) |  |  |
| Within 5 years | LGBM | 0.0748 | 0.7560 | 0.0318 | 0.1523 |
|  |  | (0.0679, 0.0844) | (0.7428, 0.7691) |  |  |
| Between 5 to 10 years | CATB | 0.1055 | 0.7234 | 0.0486 | 0.2030 |
|  |  | (0.0995, 0.1135) | (0.7133, 0.7336) |  |  |
| Within 10 years | XGB | 0.1799 | 0.7439 | 0.0955 | 0.3352 |
|  |  | (0.1713, 0.1897) | (0.7358, 0.7521) |  |  |

**Reference**

[1]	F.E. Schjerven, E.M.L. Ingeström, I. Steinsland, F. Lindseth, Development of risk models of incident hypertension using machine learning on the HUNT study data, Sci Rep 14 (2024). https://doi.org/10.1038/s41598-024-56170-7.
