# Supplementary material for: Multi-horizon machine learning for population-level hypertension risk stratification in the UK Biobank
Source: Eur Heart J Digit Health. 2026 Aug 10;7(7):ztag128. doi: 10.1093/ehjdh/ztag128 (PMC13505867; doi:10.1093/ehjdh/ztag128)
Supplement: Supplementary file 1 [file ztag128_supplementary_data.zip › Supplementary Material S7 - Variable Information.pdf]

* Note : Check Code File 'fetch_data_ukb_rap_new.ipynb' for more information

Category	Variable Name (in Analysis)	Fetched UK Biobank Variable(s) Used	Derived/Selected Variable Name(s) in Code*	Type	Values / Range / Units	Derivation Method
Personal Features	Sex	Data-Field 31: Sex	p31	Categorical (Nominal)	Female, Male	The raw numerical codes were mapped to their corresponding string representation. (0: Female, 1: Male)
	Age	Data-Field 21003: Age when attended assessment centre	p21003_i0	Numerical (Integer)	Years	The age of the participant at the initial assessment centre visit (instance 0) was used directly. This is the age truncated to a whole year.
Sociodemographic Features	Type of accommodation lived in	Data-Field 670: Type of accommodation lived in	p670_i0	Categorical (Nominal)	Permanent Housing, Temporary or Mobile Housing, Specialized or Supported Housing, None of the above	Raw codes were grouped into broader categories: 'A house or bungalow' and 'A flat, maisonette or apartment' were mapped to 'Permanent Housing'; 'Sheltered accommodation' and 'Care home' to 'Specialized or Supported Housing'; 'Mobile or temporary structure' to 'Temporary or Mobile Housing'. 'None of the above' remained as is, and 'Prefer not to answer' was treated as missing.
	Own or rent accommodation lived in	Data-Field 680: Own or rent accommodation lived in	p680_i0	Categorical (Nominal)	Own outright or living rent free, Own with a mortgage Or Paying Rent, Pay part rent and part mortgage (shared ownership), None of the above	Raw codes were grouped: 'Own outright' and 'Live in accommodation rent free' were mapped to 'Own outright or living rent free'; 'Own with a mortgage', 'Rent - from local authority', and 'Rent - from private landlord' were mapped to 'Own with a mortgage Or Paying Rent'. For participants in specialized housing, the value was set to 'None of the above'.
	Number in household	Data-Field 709: Number in household	p709_i0	Numerical (Integer)	Count of people	Raw numerical values were used directly. 'Do not know' and 'Prefer not to answer' were treated as missing. For participants in specialized housing, a minimum household number of 1 was assumed.
	Average total household income before tax	Data-Field 738: Average total household income before tax	p738_i0	Categorical (Ordinal), represented as Float	0.09, 0.195, 0.415, 0.76, 1.0	Categorical income brackets were mapped to their median values, scaled to a 0-1 range. For participants in specialized housing, a value of 0 was assumed.<br>- Sheltered/Care homes participants : 0<br>- Less than 18,000 : ((18000+0)/(2*100000)) = 0.09<br>- 18,000 to 30,999 : ((30999+8000)/(2*100000)) = 0.195<br>- 31,000 to 51,999 : ((51999+31000)/(2*100000)) = 0.415<br>- 52,000 to 100,000 : ((52000+100000)/(2*100000)) = 0.76<br>- Greater than 100,000 : 1
	Current employment status	Data-Field 6142: Current employment status<br>Data-Field 20119: Current employment status - corrected	p6142_i0_a0	Categorical (Nominal)	Employed, Not Employed	Multiple choice answers from field 6142 were combined. If any selection was 'In paid employment or self-employed', the final value was 'Employed'; otherwise, it was 'Not Employed'. This was then cross-referenced with the corrected data from field 20119, with the corrected data taking precedence.
	Qualifications	Data-Field 6138: Qualifications	p6138_i0_a0	Categorical (Ordinal) represented as Integer	0, 1, 2, 3	Multiple choice answers were mapped to a numerical hierarchy: 'None of the above' (0); 'CSEs' or 'O levels/GCSEs' (1); 'A levels/AS levels' or 'NVQ/HND/HNC' (2); 'College/University degree' or 'Other professional qualifications' (3). The highest qualification selected by a participant was used as their final value.
	Ethnic background	Data-Field 21000: Ethnic background	p21000_i0	Categorical (Nominal)	White, Mixed, Asian or Asian British, Black or Black British, Chinese, Other ethnic group	Detailed sub-categories were grouped into their top-level ethnic categories.
	Attendance/disability/mobility allowance	Data-Field 6146: Attendance/disability/mobility allowance	p6146_i0_a0	Binary (Categorical)	yes, no	Multiple choice answers were combined into a single binary variable. If a participant selected 'Attendance allowance', 'Disability living allowance', or 'Blue badge', the value was set to 'yes'; otherwise, it was set to 'no'.
Family History	Parents suffering from high blood pressure	Data-Field 20107: Illnesses of father<br>Data-Field 20110: Illnesses of mother	Parents suffering from high blood pressure	Categorical (Nominal)	both, mother, father, none, unknown	Derived by checking for 'High blood pressure' in the illness lists for both parents. 'both' if both had it; 'mother' or 'father' if only one had it; 'unknown' if data was missing for either; 'none' otherwise.
Medication	Number of treatments/medications taken	Data-Field 137: Number of treatments/medications taken	p137_i0	Numerical (Integer)	Count of medications	The raw numerical count of medications was used directly.
	Taking other prescription medications	Data-Field 2492: Taking other prescription medications	p2492_i0	Binary (Categorical)	yes, no	Raw codes were mapped to 'yes' (1) or 'no' (0).
	Vitamin and mineral supplements	Data-Field 6155: Vitamin and mineral supplements	p6155_i0_a0	Binary (Categorical)	yes, no	If any vitamin supplement was selected from the list, the value was set to 'yes'; otherwise, 'no'.
	Mineral and other dietary supplements	Data-Field 6179: Mineral and other dietary supplements	p6179_i0_a0	Binary (Categorical)	yes, no	If any mineral or other supplement was selected, the value was set to 'yes'; otherwise, 'no'.
	Cholesterol lowering medication	Data-Field 6177: Medication for cholesterol, blood pressure or diabetes (males)<br>Data-Field 6153: Medication for cholesterol, blood pressure, diabetes, or take exogenous hormones (females)	Cholesterol lowering medication	Binary (Categorical)	yes, no	Combined from two gender-specific fields. If 'Cholesterol lowering medication' was selected in either field, the value was set to 'yes'; otherwise, 'no'.
	Insulin	Data-Field 6177: Medication for cholesterol, blood pressure or diabetes (males)<br>Data-Field 6153: Medication for cholesterol, blood pressure, diabetes, or take exogenous hormones (females)	Insulin	Binary (Categorical)	yes, no	Combined from two gender-specific fields. If 'Insulin' was selected in either field, the value was set to 'yes'; otherwise, 'no'.
	Aspirin	Data-Field 6154: Medication for pain relief, constipation,	Aspirin	Binary (Categorical)	yes, no	If 'Aspirin' was selected, the value was set to 'yes'; otherwise, 'no'.
	Ibuprofen (e.g. Nurofen)	Data-Field 6154: Medication for pain relief, constipation,	Ibuprofen (e.g. Nurofen)	Binary (Categorical)	yes, no	If 'Ibuprofen' was selected, the value was set to 'yes'; otherwise, 'no'.
	Paracetamol	Data-Field 6154: Medication for pain relief, constipation,	Paracetamol	Binary (Categorical)	yes, no	If 'Paracetamol' was selected, the value was set to 'yes'; otherwise, 'no'.
	Ranitidine (e.g. Zantac)	Data-Field 6154: Medication for pain relief, constipation,	Ranitidine (e.g. Zantac)	Binary (Categorical)	yes, no	If 'Ranitidine' was selected, the value was set to 'yes'; otherwise, 'no'.
	Omeprazole (e.g. Zanprol)	Data-Field 6154: Medication for pain relief, constipation,	Omeprazole (e.g. Zanprol)	Binary (Categorical)	yes, no	If 'Omeprazole' was selected, the value was set to 'yes'; otherwise, 'no'.
	Laxatives (e.g. Dulcolax, Senokot)	Data-Field 6154: Medication for pain relief, constipation,	Laxatives (e.g. Dulcolax, Senokot)	Binary (Categorical)	yes, no	If 'Laxatives' was selected, the value was set to 'yes'; otherwise, 'no'.

Psychosocial Factors	Frequency of friend/family visits	Data-Field 1031: Frequency of friend/family visits	p1031_i0	Categorical (Ordinal), represented as Float	Range: 0 to 1	Categorical frequencies were mapped to a yearly scale: 'Almost daily' (1), '2-4 times a week' (0.43), 'About once a week' (0.14), 'About once a month' (0.033), 'Once every few months' (0.016), 'Never' or 'No friends/family outside household' (0).<br>- Almost daily : (365/365) = 1<br>- 2-4 times a week : ((3*52)/365) = 0.43<br>- About once a week : ((1*52)/365) = 0.14<br>- About once a month : (12/365) = 0.033<br>- Once every few months : (6/365) = 0.016<br>- Never or almost never = 0<br>- No friends/family outside household = 0
	Leisure/social activities	Data-Field 6160: Leisure/social activities	p6160_i0_a0	Binary (Categorical)	yes, no	If any activity (e.g., 'Sports club or gym', 'Pub or social club') was selected, the value was set to 'yes'; otherwise, 'no'.
	Able to confide	Data-Field 2110: Able to confide in someone close to you	p2110_i0	Categorical (Ordinal), represented as Float	Range: 0 to 1	Categorical frequencies were mapped to a yearly scale: 'Almost daily' (1), '2-4 times a week' (0.43), 'About once a week' (0.14), 'About once a month' (0.033), 'Once every few months' (0.016), 'Never or almost never' (0).<br>- Almost daily : (365/365) = 1<br>- 2-4 times a week : ((3*52)/365) = 0.43<br>- About once a week : ((1*52)/365) = 0.14<br>- About once a month : (12/365) = 0.033<br>- Once every few months : (6/365) = 0.016<br>- Never or almost never = 0
	Neuroticism score	Data-Field 20127: Neuroticism score	p20127_i0	Numerical (Integer)	Range: 0 to 12	The summary score, representing the count of 'Yes' answers to 12 neurotic behaviour questions, was used directly.
	Risk taking	Data-Field 2040: Risk taking	p2040_i0	Binary (Categorical)	yes, no	Raw codes were mapped to 'yes' (1) or 'no' (0).
	Frequency of depressed mood in last 2 weeks	Data-Field 2050: Frequency of depressed mood in last 2 weeks	p2050_i0	Categorical (Ordinal), represented as Float	Range: 0 to 1	Categorical frequencies were mapped to a scaled value: 'Not at all' (0), 'Several days' (0.35), 'More than half the days' (0.65), 'Nearly every day' (1).<br>- Not at all = 0<br>- Several days : (0+0.35) = 0.35<br>- More than half the days : (1-0.35) = 0.65<br>- Nearly every day = 1
	Frequency of unenthusiasm / disinterest in last 2 weeks	Data-Field 2060: Frequency of unenthusiasm / disinterest in last 2 weeks	p2060_i0	Categorical (Ordinal), represented as Float	Range: 0 to 1	Categorical frequencies were mapped to a scaled value (same as above): 'Not at all' (0), 'Several days' (0.35), 'More than half the days' (0.65), 'Nearly every day' (1).
	Frequency of tenseness / restlessness in last 2 weeks	Data-Field 2070: Frequency of tenseness / restlessness in last 2 weeks	p2070_i0	Categorical (Ordinal), represented as Float	Range: 0 to 1	Categorical frequencies were mapped to a scaled value (same as above): 'Not at all' (0), 'Several days' (0.35), 'More than half the days' (0.65), 'Nearly every day' (1).
	Frequency of tiredness / lethargy in last 2 weeks	Data-Field 2080: Frequency of tiredness / lethargy in last 2 weeks	p2080_i0	Categorical (Ordinal), represented as Float	Range: 0 to 1	Categorical frequencies were mapped to a scaled value (same as above): 'Not at all' (0), 'Several days' (0.35), 'More than half the days' (0.65), 'Nearly every day' (1).
	Illness, injury, bereavement, stress in last 2 years	Data-Field 6145: Illness, injury, bereavement, stress in last 2 years	p6145_i0_a0	Binary (Categorical)	yes, no	If any stressful event was selected from the list, the value was set to 'yes'; otherwise, 'no'.
	Seen doctor (GP) or psychiatrist for nerves, anxiety, tension or depression	Data-Field 2090: Seen doctor (GP) for nerves, anxiety, tension or depression<br>Data-Field 2100: Seen a psychiatrist for nerves, anxiety, tension or depression	Seen doctor (GP) or psychiatrist for nerves, anxiety, tension or depression	Binary (Categorical)	yes, no	If a participant answered 'yes' to seeing either a GP or a psychiatrist for these issues, the combined variable was set to 'yes'; otherwise, 'no'.
Environmental Factors	Time spend outdoors in summer	Data-Field 1050: Time spend outdoors in summer	p1050_i0	Numerical (Float)	hours/day	Raw values were used. 'Less than an hour a day' (-10) was mapped to 0.5.
	Time spend outdoors in winter	Data-Field 1060: Time spend outdoors in winter	p1060_i0	Numerical (Float)	hours/day	Raw values were used. 'Less than an hour a day' (-10) was mapped to 0.5.
	Exposure to tobacco smoke	Data-Field 1269: Exposure to tobacco smoke at home<br>Data-Field 1279: Exposure to tobacco smoke outside home	Exposure to tobacco smoke	Numerical (Float)	hours/week	The hours per week of exposure at home and outside the home were summed.
Smoking	Current smoker	Data-Field 20116: Smoking status	Current smoker	Binary (Categorical)	yes, no	Derived from the summary 'Smoking status' field. If status was 'Current', the value was 'yes'; otherwise, 'no'.
	Pack years of smoking	Data-Field 20161: Pack years of smoking	p20161_i0	Numerical (Float)	pack-years	UK Biobank pre-calculated value used: (cigarettes per day / 20) x (age stopped - age started), with an additional 0.5-year subtraction for those who quit for >6 months. Values -1 and -3 were mapped to NaN. For non-smokers and light smokers (Tobacco smoking status of 0 = No, 1 = Tried once or twice, or 2 = Occasionally), the value was set to 0.
	Tobacco smoking status	Data-Field 1239: Current tobacco smoking<br>Data-Field 1249: Past tobacco smoking	Tobacco smoking status	Categorical (Ordinal) represented as Integer	0, 1, 2, 3	Combined from current and past smoking habits and mapped to an ordinal scale: 'No' (0), 'Tried once or twice' (1), 'Occasionally' (2), 'Most or all days' (3).
Alcohol	Alcohol drinker status	Data-Field 20117: Alcohol drinker status	p20117_i0	Categorical (Nominal)	Current, Previous, Never	Raw codes were mapped to their string representations.
	Alcohol average weekly intake	Data-Field 4407: Average monthly red wine intake<br>Data-Field 1568: Average weekly red wine intake<br>Data-Field 4418: Average monthly champagne plus white wine intake<br>Data-Field 1578: Average weekly champagne plus white wine intake<br>Data-Field 4429: Average monthly beer plus cider intake<br>Data-Field 1588: Average weekly beer plus cider intake<br>Data-Field 4440: Average monthly spirits intake<br>Data-Field 1598: Average weekly spirits intake<br>Data-Field 4451: Average monthly fortified wine intake<br>Data-Field 1608: Average weekly fortified wine intake<br>Data-Field 4462: Average monthly intake of other alcoholic	Alcohol average weekly intake	Numerical (Float)	alcohol units/week	Weekly alcohol units were derived by summing intake across all beverage types (red wine, white wine/champagne, beer/cider, spirits, fortified wine, and other alcoholic drinks). Monthly intakes were divided by 4 to convert to weekly. Each beverage count was multiplied by its per-serving alcohol unit weight before summing: red wine = 2.2, white wine/champagne = 2.2, beer/cider = 2.5, spirits = 1.2, fortified wine = 1.0, other alcoholic drinks = 1.3 (mean values per NHS guidance). Values of -1 (Do not know) and -3 (Prefer not to answer) were set to NaN. Final value = sum of (all weekly columns) + (monthly columns * unit / 4).

	Alcohol intake frequency	Data-Field 1558: Alcohol intake frequency	p1558_i0	Categorical (Ordinal), represented as Float	Range: 0 to 1	Categorical frequencies were mapped to a yearly scale: 'Daily or almost daily' (1), 'Three or four times a week' (0.50), 'Once or twice a week' (0.21), 'One to three times a month' (0.066), 'Special occasions only' (0.016), 'Never' (0).<br>- Daily or almost daily : (365/365) = 1<br>- Three or four times a week : ((3.5*52)/365) = 0.50<br>- Once or twice a week : ((1.5*52)/365) = 0.21<br>- One to three times a month : ((2*12)/365) = 0.066<br>- Special occasions only : (6/365) = 0.016<br>- Never = 0
**Sleep Features**	Sleep duration	Data-Field 1160: Sleep duration	p1160_i0	Numerical (Integer)	hours/day	The raw numerical value for hours of sleep per 24 hours (including naps) was used.
	Sleeplessness / insomnia	Data-Field 1200: Sleeplessness / insomnia	p1200_i0	Categorical (Ordinal) represented as Integer	1,2,3	Coded responses were used directly: 'Never/rarely' (1), 'Sometimes' (2), 'Usually' (3).
**Physical Activity**	Usual walking pace	Data-Field 924: Usual walking pace	p924_i0	Categorical (Ordinal), represented as Float	0, 2.5, 3.5, 4.5 (mph)	Categorical paces were mapped to their approximate speed in miles per hour: 'Slow' (2.5), 'Steady average' (3.5), 'Brisk' (4.5), 'None of the above' (0).<br>- Slow pace : (less than 3 miles/hr) = 2.5<br>- Steady average pace : (between than 3-4 miles/hr) = 3.5<br>- Brisk pace : (more than 4 miles/hr) = 4.5<br>- None of the above = 0
	Frequency of stair climbing in last 4 weeks	Data-Field 943: Frequency of stair climbing in last 4 weeks	p943_i0	Numerical (Integer)	times/day	Categorical frequency ranges were mapped to their median values (e.g., '1-5 times a day' became 3).<br>- None : 0<br>- 1-5 times a day : (5+1)//2 = 3<br>- 6-10 times a day : (10+6)//2 = 8<br>- 11-15 times a day : (15+11)//2 = 13<br>- 16-20 times a day : (20+16)//2 = 18<br>- More than 20 times a day : (25+21)//2 = 23
	Duration of walks per week	Data-Field 864: Number of days/week walked 10+ minutes<br>Data-Field 874: Duration of walks	Duration of walks per week	Numerical (Float)	minutes/week	Calculated by multiplying the number of days walked per week by the duration of walks per day. Missing values were imputed with the median before multiplication. Unable to walk assumed as 0.
	Duration of moderate activity per week	Data-Field 884: Number of days/week of moderate physical activity 10+ minutes<br>Data-Field 894: Duration of moderate activity	Duration of moderate activity per week	Numerical (Float)	minutes/week	Calculated by multiplying the number of days of moderate activity per week by the duration per day. Missing values were imputed with the median before multiplication. Unable to walk assumed as 0.
	Duration of vigorous activity per week	Data-Field 904: Number of days/week of vigorous physical activity 10+ minutes<br>Data-Field 914: Duration of vigorous activity	Duration of vigorous activity per week	Numerical (Float)	minutes/week	Calculated by multiplying the number of days of vigorous activity per week by the duration per day. Missing values were imputed with the median before multiplication. Unable to walk assumed as 0.
	Duration of sedentary activity per week	Data-Field 1090: Time spent driving<br>Data-Field 1080: Time spent using computer<br>Data-Field 1070: Time spent watching television (TV)	Duration of sedentary activity per week	Numerical (Float)	minutes/week	The daily hours spent on driving, computer use, and watching TV were summed, converted to minutes, and then multiplied by 7 to get a weekly total.<br>- Time spent driving * 60 + Time spent using computer * 60 + Time spent watching television (TV) * 60) * 7
	Duration of Heavy DIY in last 4 weeks	Data-Field 2624: Frequency of heavy DIY in last 4 weeks<br>Data-Field 2634: Duration of heavy DIY	Duration of Heavy DIY in last 4 weeks	Numerical (Float)	minutes	Calculated by multiplying the frequency of heavy DIY in the last 4 weeks by the duration of each session. Categorical frequency and duration ranges were first mapped to numerical estimates.
	Duration of Light DIY in last 4 weeks	Data-Field 1011: Frequency of light DIY in last 4 weeks<br>Data-Field 1021: Duration of light DIY	Duration of Light DIY in last 4 weeks	Numerical (Float)	minutes	Calculated by multiplying the frequency of light DIY in the last 4 weeks by the duration of each session. Categorical frequency and duration ranges were first mapped to numerical estimates.
	Duration of Other exercises in last 4 weeks	Data-Field 3637: Frequency of other exercises in last 4 weeks<br>Data-Field 3647: Duration of other exercises	Duration of Other exercises in last 4 weeks	Numerical (Float)	minutes	Calculated by multiplying the frequency of other exercises in the last 4 weeks by the duration of each session. Categorical frequency and duration ranges were first mapped to numerical estimates.
	Duration of Strenuous sports in last 4 weeks	Data-Field 991: Frequency of strenuous sports in last 4 weeks<br>Data-Field 1001: Duration of strenuous sports	Duration of Strenuous sports in last 4 weeks	Numerical (Float)	minutes	Calculated by multiplying the frequency of strenuous sports in the last 4 weeks by the duration of each session. Categorical frequency and duration ranges were first mapped to numerical estimates.
	Duration of Walking for pleasure in last 4 weeks	Data-Field 971: Frequency of walking for pleasure in last 4 weeks<br>Data-Field 981: Duration walking for pleasure	Duration of Walking for pleasure in last 4 weeks	Numerical (Float)	minutes	Calculated by multiplying the frequency of walking for pleasure in the last 4 weeks by the duration of each session. Categorical frequency and duration ranges were first mapped to numerical estimates.
	Cooked vegetable intake	Data-Field 1289: Cooked vegetable intake	p1289_i0	Numerical (Float)	tablespoons/day	Raw values were used. 'Less than one' (-10) was mapped to 0.5.
	Salad / raw vegetable intake	Data-Field 1299: Salad / raw vegetable intake	p1299_i0	Numerical (Float)	tablespoons/day	Raw values were used. 'Less than one' (-10) was mapped to 0.5.
	Fresh fruit intake	Data-Field 1309: Fresh fruit intake	p1309_i0	Numerical (Float)	pieces/day	Raw values were used. 'Less than one' (-10) was mapped to 0.5.
	Dried fruit intake	Data-Field 1319: Dried fruit intake	p1319_i0	Numerical (Float)	pieces/day	Raw values were used. 'Less than one' (-10) was mapped to 0.5.
	Bread intake	Data-Field 1438: Bread intake	p1438_i0	Numerical (Float)	slices/week	Raw values were used. 'Less than one' (-10) was mapped to 0.5.
	Cereal intake	Data-Field 1458: Cereal intake	p1458_i0	Numerical (Float)	bowls/week	Raw values were used. 'Less than one' (-10) was mapped to 0.5.
	Tea intake	Data-Field 1488: Tea intake	p1488_i0	Numerical (Float)	cups/day	Raw values were used. 'Less than one' (-10) was mapped to 0.5.
	Coffee intake	Data-Field 1498: Coffee intake	p1498_i0	Numerical (Float)	cups/day	Raw values were used. 'Less than one' (-10) was mapped to 0.5.
	Water intake	Data-Field 1528: Water intake	p1528_i0	Numerical (Float)	glasses/day	Raw values were used. 'Less than one' (-10) was mapped to 0.5.

**Diet**	Oily fish intake	Data-Field 1329: Oily fish intake	p1329_i0	Categorical (Ordinal), represented as Float	Range: 0 to 1	Categorical frequencies were mapped to a yearly scale (e.g., 'Never' to 0, 'Once or more daily' to 1).<br>Never - 0<br>Less than once a week ((1/2)*52) / 365 = 0.07<br>Once a week (1*52) / 365 = 0.14<br>2-4 times a week (3*52) / 365 = 0.43<br>5-6 times a week (5.5*52) / 365 = 0.78<br>Once or more daily 365/365 = 1
	Non-oily fish intake	Data-Field 1339: Non-oily fish intake	p1339_i0	Categorical (Ordinal), represented as Float	Range: 0 to 1	Categorical frequencies were mapped to a yearly scale (as above) (e.g., 'Never' to 0, 'Once or more daily' to 1).
	Processed meat intake	Data-Field 1349: Processed meat intake	p1349_i0	Categorical (Ordinal), represented as Float	Range: 0 to 1	Categorical frequencies were mapped to a yearly scale (as above) (e.g., 'Never' to 0, 'Once or more daily' to 1).
	Poultry intake	Data-Field 1359: Poultry intake	p1359_i0	Categorical (Ordinal), represented as Float	Range: 0 to 1	Categorical frequencies were mapped to a yearly scale (as above) (e.g., 'Never' to 0, 'Once or more daily' to 1).
	Beef intake	Data-Field 1369: Beef intake	p1369_i0	Categorical (Ordinal), represented as Float	Range: 0 to 1	Categorical frequencies were mapped to a yearly scale (as above) (e.g., 'Never' to 0, 'Once or more daily' to 1).
	Lamb/mutton intake	Data-Field 1379: Lamb/mutton intake	p1379_i0	Categorical (Ordinal), represented as Float	Range: 0 to 1	Categorical frequencies were mapped to a yearly scale (as above) (e.g., 'Never' to 0, 'Once or more daily' to 1).
	Pork intake	Data-Field 1389: Pork intake	p1389_i0	Categorical (Ordinal), represented as Float	Range: 0 to 1	Categorical frequencies were mapped to a yearly scale (as above) (e.g., 'Never' to 0, 'Once or more daily' to 1).
	Cheese intake	Data-Field 1408: Cheese intake	p1408_i0	Categorical (Ordinal), represented as Float	Range: 0 to 1	Categorical frequencies were mapped to a yearly scale (as above) (e.g., 'Never' to 0, 'Once or more daily' to 1).
	Salt added to food	Data-Field 1478: Salt added to food	p1478_i0	Categorical (Ordinal) represented as Integer	1, 2, 3, 4	Coded responses were used directly: 'Never/rarely' (1), 'Sometimes' (2), 'Usually' (3), 'Always' (4).
	Milk type used	Data-Field 1418: Milk type used	p1418_i0	Categorical (Nominal)	High fat dairy, Low fat dairy, Soya, Other, None	Raw codes were grouped into broader categories. 'Full cream' mapped to 'High fat dairy'; 'Semi-skimmed' and 'Skimmed' to 'Low fat dairy'.
	Coffee type	Data-Field 1508: Coffee type	p1508_i0	Categorical (Nominal)	Contains Caffeine, Decaffeinated, None	Raw codes were grouped into 'Contains Caffeine' and 'Decaffeinated'. If coffee intake was 0, it was coded as 'None'.
	Cereal type	Data-Field 1468: Cereal type	p1468_i0	Categorical (Nominal)	Whole Grain & High-Fiber Cereals, Refined & Processed Cereals, None	Raw codes were grouped. 'Bran', 'Biscuit', 'Oat', and 'Muesli' cereals mapped to 'Whole Grain & High-Fiber Cereals'; 'Other' mapped to 'Refined & Processed Cereals'. If cereal intake was 0, it was coded as 'None'.
	Bread type	Data-Field 1448: Bread type	p1448_i0	Categorical (Nominal)	Whole Grain & Less Processed Bread, Refined flour or other bread, None	Raw codes were grouped. 'Brown' and 'Wholemeal' mapped to 'Whole Grain & Less Processed Bread'; 'White' and 'Other' to 'Refined flour or other bread'. If bread intake was 0, it was coded as 'None'.
	Spread type	Data-Field 1428: Spread type	p1428_i0	Categorical (Nominal)	Butter, Non Butter, Never/rarely use spread	Raw codes were grouped. 'Butter/spreadable butter' mapped to 'Butter'; 'Flora Pro-Active/Benecol' and 'Other' mapped to 'Non Butter'.
**Cardiovascular Assessment**	Diastolic blood pressure, automated reading	Data-Field 4079: Diastolic blood pressure, automated reading	p4079_i0_a1	Numerical (Integer)	mmHg	The minimum of the of two automated readings was used.
	Systolic blood pressure, automated reading	Data-Field 4080: Systolic blood pressure, automated reading	p4080_i0_a1	Numerical (Integer)	mmHg	The minimum of the of two automated readings was used.
	Pulse rate, automated reading	Data-Field 102: Pulse rate, automated reading	p102_i0_a1	Numerical (Integer)	bpm	The minimum of the of two automated readings was used.
**Physical Measures**	Hand grip strength (left)	Data-Field 46: Hand grip strength (left)	p46_i0	Numerical (Float)	kg	Raw measurement was used directly.
	Hand grip strength (right)	Data-Field 47: Hand grip strength (right)	p47_i0	Numerical (Float)	kg	Raw measurement was used directly.
	Standing height	Data-Field 50: Standing height	p50_i0	Numerical (Float)	cm	Raw measurement was used directly.
	Weight	Data-Field 21002: Weight	p21002_i0	Numerical (Float)	kg	Raw measurement was used directly.
	Body mass index (BMI)	Data-Field 21001: Body mass index (BMI)	p21001_i0	Numerical (Float)	kg/m^2	Pre-calculated BMI from the dataset was used directly.
**Blood Biomarkers**	Red blood cell (erythrocyte) count	Data-Field 30010: Red blood cell (erythrocyte) count	p30010_i0	Numerical (Float)	$x10^{12}$ cells/L	Raw measurement was used directly.
	Haemoglobin concentration	Data-Field 30020: Haemoglobin concentration	p30020_i0	Numerical (Float)	g/dL	Raw measurement was used directly.
	Albumin	Data-Field 30600: Albumin	p30600_i0	Numerical (Float)	g/L	Raw measurement was used directly.
	Apolipoprotein A	Data-Field 30630: Apolipoprotein A	p30630_i0	Numerical (Float)	g/L	Raw measurement was used directly.
	Apolipoprotein B	Data-Field 30640: Apolipoprotein B	p30640_i0	Numerical (Float)	g/L	Raw measurement was used directly.
	Direct bilirubin	Data-Field 30660: Direct bilirubin	p30660_i0	Numerical (Float)	umol/L	Raw measurement was used directly.
	Total bilirubin	Data-Field 30840: Total bilirubin	p30840_i0	Numerical (Float)	umol/L	Raw measurement was used directly.
	Cholesterol	Data-Field 30690: Cholesterol	p30690_i0	Numerical (Float)	mmol/L	Raw measurement was used directly.
	HDL cholesterol	Data-Field 30760: HDL cholesterol	p30760_i0	Numerical (Float)	mmol/L	Raw measurement was used directly.
	Creatinine	Data-Field 30700: Creatinine	p30700_i0	Numerical (Float)	umol/L	Raw measurement was used directly.
	C-reactive protein	Data-Field 30710: C-reactive protein	p30710_i0	Numerical (Float)	mg/L	Raw measurement was used directly.
	Lipoprotein A	Data-Field 30790: Lipoprotein A	p30790_i0	Numerical (Float)	nmol/L	Raw measurement was used directly.
	Triglycerides	Data-Field 30870: Triglycerides	p30870_i0	Numerical (Float)	mmol/L	Raw measurement was used directly.
	Vitamin D	Data-Field 30890: Vitamin D	p30890_i0	Numerical (Float)	nmol/L	Raw measurement was used directly.
**Multimorbidity History**	Asthma, ... [38 other conditions]	Multiple date fields corresponding to first occurrence of each condition (e.g., Data-Field 131494 for Asthma)	p131494, etc.	Binary (Categorical)	yes, no	For each of the 39 conditions (e.g., Asthma, Diabetes, Stroke), the date of first occurrence was fetched. A new binary variable was created for each condition. The value was set to 'yes' if the condition was reported on or before the participant's initial assessment visit date (p53_i0), and 'no' otherwise.
**Dependant/Target Variable**	Hypertension Risk	Data-Field 131286: Date I10 first reported (essential (primary) hypertension)	Hypertension Risk	Datetime	Date	The date of the first reported occurrence of essential (primary) hypertension (ICD-10 code I10) was used. This date along with visit date was used to derive the dependent variables.
