# Supplementary material for: Multi-horizon machine learning for population-level hypertension risk stratification in the UK Biobank
Source: Eur Heart J Digit Health. 2026 Aug 10;7(7):ztag128. doi: 10.1093/ehjdh/ztag128 (PMC13505867; doi:10.1093/ehjdh/ztag128)
Supplement: Supplementary file 1 [file ztag128_supplementary_data.zip › Supplementary Material S8 - Multimorbidity Info.pdf]

* Note : Participants with history of Hypertensive Disorder are eliminated

Condition (Multimorbidity History)	ICD10 Code	Health Outcome Definition	UK BioBank Category	UK BioBank FieldID
**Hypertensive Disorders***	I11	hypertensive heart disease	Circulatory system disorders	131288
	I12	hypertensive renal disease	Circulatory system disorders	131290
	I13	hypertensive heart and renal disease	Circulatory system disorders	131292
	I15	secondary hypertension	Circulatory system disorders	131294
	O10	pre-existing hypertension complicating pregnancy, childbirth and the puerperium	Pregnancy, childbirth and the puerperium	132180
	O11	pre-existing hypertensive disorder with superimposed proteinuria	Pregnancy, childbirth and the puerperium	132182
	O13	gestational [pregnancy-induced] hypertension without significant proteinuria	Pregnancy, childbirth and the puerperium	132186
	O14	gestational [pregnancy-induced] hypertension with significant proteinuria	Pregnancy, childbirth and the puerperium	132188
	O15	eclampsia	Pregnancy, childbirth and the puerperium	132190
	O16	unspecified maternal hypertension	Pregnancy, childbirth and the puerperium	132192
	I27.0	primary pulmonary hypertension	Circulatory system disorders	131310
	K76.6	portal hypertension	Digestive system disorders	131670
**Asthma**	J45	asthma	Respiratory system disorders	131494
**Depression**	F31	bipolar affective disorder (manic depression)	Mental and behavioural disorders	130892
	F32	depressive episode	Mental and behavioural disorders	130894
	F33	recurrent depressive disorder	Mental and behavioural disorders	130896
	F34	persistent mood [affective] disorders	Mental and behavioural disorders	130898
	F92.0	mixed disorder of conduct and emotions (depressive conduct disorder)	Mental and behavioural disorders	130980
**Diabetes**	E10	insulin-dependent diabetes mellitus	Endocrine, nutritional and metabolic disorders	130706
	E11	non-insulin-dependent diabetes mellitus	Endocrine, nutritional and metabolic disorders	130708
	E12	malnutrition-related diabetes mellitus	Endocrine, nutritional and metabolic disorders	130710
	E13	other specified diabetes mellitus	Endocrine, nutritional and metabolic disorders	130712
	E14	unspecified diabetes mellitus	Endocrine, nutritional and metabolic disorders	130714
**Angina**	I20	angina pectoris	Circulatory system disorders	131296
**Migraine**	G43	migraine	Nervous system disorders	131052
**Eczema or dermatitis**	L20	atopic dermatitis	Skin and subcutaneous tissue disorders	131720
	L21	seborrhoeic dermatitis	Skin and subcutaneous tissue disorders	131722
	L22	diaper [napkin] dermatitis	Skin and subcutaneous tissue disorders	131724
	L23	allergic contact dermatitis	Skin and subcutaneous tissue disorders	131726
	L24	irritant contact dermatitis	Skin and subcutaneous tissue disorders	131728
	L25	unspecified contact dermatitis	Skin and subcutaneous tissue disorders	131730
	L26	exfoliative dermatitis	Skin and subcutaneous tissue disorders	131732
	L27	dermatitis due to substances taken internally	Skin and subcutaneous tissue disorders	131734
	L28	lichen simplex chronicus and prurigo (circumscribed neurodermatitis)	Skin and subcutaneous tissue disorders	131736
	L29	pruritus (itch)	Skin and subcutaneous tissue disorders	131738
	L30	other dermatitis	Skin and subcutaneous tissue disorders	131740
**Irritable bowel syndrome**	K58	irritable bowel syndrome	Digestive system disorders	131638
**Myocardial Infarction**	I21	acute myocardial infarction	Circulatory system disorders	131298
	I22	subsequent myocardial infarction	Circulatory system disorders	131300
**Osteoporosis**	M80	osteoporosis with pathological fracture	Musculoskeletal system and connective tissue disorders	131962
	M81	osteoporosis without pathological fracture	Musculoskeletal system and connective tissue disorders	131964
	M82	osteoporosis in diseases classified elsewhere	Musculoskeletal system and connective tissue disorders	131966
**Stroke**	I60	subarachnoid haemorrhage	Circulatory system disorders	131360
	I61	intracerebral haemorrhage	Circulatory system disorders	131362
	I63	cerebral infarction	Circulatory system disorders	131366
	I63.9	ischaemic stroke (another field - date of ischaemic stroke)	Circulatory system disorders	42008
	I64	stroke, not specified as haemorrhage or infarction	Circulatory system disorders	131368
**Anxiety or panic attacks**	F40	phobic anxiety disorders	Mental and behavioural disorders	130904
	F41	other anxiety disorders	Mental and behavioural disorders	130906
	F43.0	Acute stress reaction (panic attack)	Mental and behavioural disorders	130910
	F93	emotional (anxiety) disorders with onset specific to childhood	Mental and behavioural disorders	130982
**Arthritis**	M05	seropositive rheumatoid arthritis	Musculoskeletal system and connective tissue disorders	131848
	M06	other rheumatoid arthritis	Musculoskeletal system and connective tissue disorders	131850
	M45	ankylosing spondylitis	Musculoskeletal system and connective tissue disorders	131912
	M15	polyarthrosis	Musculoskeletal system and connective tissue disorders	131868
	M16	coxarthrosis [arthrosis of hip]	Musculoskeletal system and connective tissue disorders	131870
	M17	gonarthrosis [arthrosis of knee]	Musculoskeletal system and connective tissue disorders	131872
	M18	arthrosis of first carpometacarpal joint	Musculoskeletal system and connective tissue disorders	131874
	M19	other arthrosis	Musculoskeletal system and connective tissue disorders	131876
	M47	spondylosis	Musculoskeletal system and connective tissue disorders	131916
	M07	psoriatic and enteropathic arthropathies	Musculoskeletal system and connective tissue disorders	131852
	M08	juvenile arthritis	Musculoskeletal system and connective tissue disorders	131854
	M09	juvenile arthritis in diseases classified elsewhere	Musculoskeletal system and connective tissue disorders	131856
	M13	other arthritis	Musculoskeletal system and connective tissue disorders	131864
	M14.3	lipoid dermatoarthritis	Musculoskeletal system and connective tissue disorders	131866
**Glaucoma**	H40	glaucoma	Eye and adnexa disorders	131186
	H42	glaucoma in diseases classified elsewhere	Eye and adnexa disorders	131188
**Epilepsy**	G40	epilepsy	Nervous system disorders	131048
**Vestibular disorder**	H81	disorders of vestibular function	Ear and mastoid process disorders	131252
**Atrial fibrillation**	I48	atrial fibrillation and flutter	Circulatory system disorders	131350
**Chronic sinusitis**	J32	chronic sinusitis	Respiratory system disorders	131468
**Tuberculosis**	A15	respiratory tuberculosis, bacteriologically and histologically confirmed	Certain infectious and parasitic diseases	130020
	A16	respiratory tuberculosis, not confirmed bacteriologically or histologically	Certain infectious and parasitic diseases	130022
	A17	tuberculosis of nervous system	Certain infectious and parasitic diseases	130024
	A18	tuberculosis of other organs	Certain infectious and parasitic diseases	130026
	A19	miliary tuberculosis	Certain infectious and parasitic diseases	130028
	G00	bacterial meningitis, not elsewhere classified	Nervous system disorders	130992
	G01	meningitis in bacterial diseases classified elsewhere	Nervous system disorders	130994

**Meningitis**	G02	meningitis in other infectious and parasitic diseases classified elsewhere	Nervous system disorders	130996
	G03	meningitis due to other and unspecified causes	Nervous system disorders	130998
	A87	viral meningitis	Certain infectious and parasitic diseases	130148
**Chronic Obstructive Pulmonary Disease (COPD)**	J44	other chronic obstructive pulmonary disease	Respiratory system disorders	131492
**Multiple Sclerosis**	G35	multiple sclerosis	Nervous system disorders	131042
**Thyroid problem**	E00	congenital iodine-deficiency syndrome	Endocrine, nutritional and metabolic disorders	130690
	E01	iodine-deficiency-related thyroid disorders and allied conditions	Endocrine, nutritional and metabolic disorders	130692
	E02	subclinical iodine-deficiency hypothyroidism	Endocrine, nutritional and metabolic disorders	130694
	E03	other hypothyroidism	Endocrine, nutritional and metabolic disorders	130696
	E04	other non-toxic goitre	Endocrine, nutritional and metabolic disorders	130698
	E05	thyrotoxicosis [hyperthyroidism]	Endocrine, nutritional and metabolic disorders	130700
	E06	thyroiditis	Endocrine, nutritional and metabolic disorders	130702
	E07	other disorders of thyroid	Endocrine, nutritional and metabolic disorders	130704
**Parkinson's disease**	G20	parkinson's disease	Nervous system disorders	131022
	G22	parkinsonism in diseases classified elsewhere	Nervous system disorders	131026
**Prostate problem**	N40	hyperplasia of prostate	Genitourinary system disorders	132072
	N41	inflammatory diseases of prostate	Genitourinary system disorders	132074
	N42	other disorders of prostate	Genitourinary system disorders	132076
**Bronchiectasis**	J47	bronchiectasis	Respiratory system disorders	131498
**Anaemia**	D50	iron deficiency anaemia	Blood, blood-forming organs and certain immune disorders	130622
	D51	vitamin B12 deficiency anaemia	Blood, blood-forming organs and certain immune disorders	130624
	D52	folate deficiency anaemia	Blood, blood-forming organs and certain immune disorders	130626
	D53	other nutritional anaemias	Blood, blood-forming organs and certain immune disorders	130628
	D55	anaemia due to enzyme disorders	Blood, blood-forming organs and certain immune disorders	130630
	D56	thalassaemia	Blood, blood-forming organs and certain immune disorders	130632
	D57	sickle-cell disorders (anaemia with/without crisis)	Blood, blood-forming organs and certain immune disorders	130634
	D58	other hereditary haemolytic anaemias	Blood, blood-forming organs and certain immune disorders	130636
	D59	acquired haemolytic anaemia	Blood, blood-forming organs and certain immune disorders	130638
	D60	acquired pure red cell aplasia [erythroblastopenia] (type of aplastic anemia)	Blood, blood-forming organs and certain immune disorders	130640
	D61	other aplastic anaemias	Blood, blood-forming organs and certain immune disorders	130642
	D62	acute posthaemorrhagic anaemia	Blood, blood-forming organs and certain immune disorders	130644
	D63	anaemia in chronic diseases classified elsewhere	Blood, blood-forming organs and certain immune disorders	130646
	D64	other anaemias	Blood, blood-forming organs and certain immune disorders	130648
**Peripheral vascular disease**	I73	other peripheral vascular diseases	Circulatory system disorders	131386
**Heart failure**	I50	heart failure	Circulatory system disorders	131354
**Cirrhosis**	K74	fibrosis and cirrhosis of liver	Digestive system disorders	131666
**Hepatitis**	B15	acute hepatitis A	Certain infectious and parasitic diseases	130194
	B16	acute hepatitis B	Certain infectious and parasitic diseases	130196
	B17	other acute viral hepatitis	Certain infectious and parasitic diseases	130198
	B18	chronic viral hepatitis	Certain infectious and parasitic diseases	130200
	B19	unspecified viral hepatitis	Certain infectious and parasitic diseases	130202
	K73	chronic hepatitis, not elsewhere classified	Digestive system disorders	131664
	K75	Nonspecific reactive hepatitis (K75.2), Granulomatous hepatitis, not elsewhere classified (K75.3), Autoimmune hepatitis (K75.4)	Digestive system disorders	131668
	B25.1	cytomegaloviral hepatitis (cytomegaloviral disease)	Certain infectious and parasitic diseases	130214
**Schizophrenia**	F20	schizophrenia	Mental and behavioural disorders	130874
**Dementia**	F00	dementia in Alzheimer's disease	Mental and behavioural disorders	130836
	F01	vascular dementia	Mental and behavioural disorders	130838
	F02	dementia in other diseases classified elsewhere	Mental and behavioural disorders	130840
	F03	unspecified dementia	Mental and behavioural disorders	130842
**Chronic kidney disease**	N18	chronic kidney disease (Stage 1-5, unspecified CKD)	Genitourinary system disorders	132032
**Inflammatory bowel disease**	K50	Crohn's disease [regional enteritis]	Digestive system disorders	131626
	K51	ulcerative colitis	Digestive system disorders	131628
**Obesity**	E66	obesity	Endocrine, nutritional and metabolic disorders	130792
**Visual disturbances and blindness**	H53	visual disturbances	Eye and adnexa disorders	131210
	H54	Visual impairment including blindness (binocular or monocular)	Eye and adnexa disorders	131212
**Other Heart Diseases**	I05	rheumatic mitral valve diseases	Circulatory system disorders	131276
	I06	rheumatic aortic valve diseases	Circulatory system disorders	131278
	I07	rheumatic tricuspid valve diseases	Circulatory system disorders	131280
	I08	multiple valve diseases	Circulatory system disorders	131282
	I09	other rheumatic heart diseases	Circulatory system disorders	131284
	I23	certain current complications following acute myocardial infarction	Circulatory system disorders	131302
	I24	other acute ischaemic heart diseases	Circulatory system disorders	131304
	I25	chronic ischaemic heart disease (Includes I25.2 - old/healed myocardial infarction)	Circulatory system disorders	131306
	I30	acute pericarditis	Circulatory system disorders	131314
	I31	other diseases of pericardium	Circulatory system disorders	131316
	I32	pericarditis in diseases classified elsewhere	Circulatory system disorders	131318
	I33	acute and subacute endocarditis	Circulatory system disorders	131320
	I34	nonrheumatic mitral valve disorders	Circulatory system disorders	131322
	I35	nonrheumatic aortic valve disorders	Circulatory system disorders	131324
	I36	nonrheumatic tricuspid valve disorders	Circulatory system disorders	131326
	I37	pulmonary valve disorders	Circulatory system disorders	131328
	I38	endocarditis, valve unspecified	Circulatory system disorders	131330
	I39	endocarditis and heart valve disorders in diseases classified elsewhere	Circulatory system disorders	131332
	I40	acute myocarditis	Circulatory system disorders	131334
	I41	myocarditis in diseases classified elsewhere	Circulatory system disorders	131336
	I42	cardiomyopathy	Circulatory system disorders	131338
	I43	cardiomyopathy in diseases classified elsewhere	Circulatory system disorders	131340
	I44	atrioventricular and left bundle-branch block	Circulatory system disorders	131342
	I45	other conduction disorders	Circulatory system disorders	131344

	I46	cardiac arrest	Circulatory system disorders	131346
	I47	paroxysmal tachycardia	Circulatory system disorders	131348
	I49	other cardiac arrhythmias	Circulatory system disorders	131352
	I51	complications and ill-defined descriptions of heart disease	Circulatory system disorders	131356
	I52	other heart disorders in diseases classified elsewhere	Circulatory system disorders	131358
**Cancer**	C00-D48	Cancer (Pregnancy, childbirth and the puerperium, Neoplasms)	Cancer	40005
